# Identification of Differentially Expressed Genes Reveal Conserved Mechanisms in the Rice-*Magnaporthe oryzae* Interaction

**DOI:** 10.3389/fpls.2022.723356

**Published:** 2022-04-05

**Authors:** Dong Liang, Zhongqiang Qi, Yan Du, Junjie Yu, Mina Yu, Rongsheng Zhang, Huijuan Cao, Xiayan Pan, Junqing Qiao, Tianqiao Song, Youzhou Liu, Zhiyi Chen, Yongfeng Liu

**Affiliations:** Institute of Plant Protection, Jiangsu Academy of Agricultural Sciences (JAAS), Nanjing, China

**Keywords:** rice-*Magnaporthe oryzae* interaction, comparative transcriptome, universal stress proteins, light-harvesting chlorophyll a/b-binding proteins, AA9 proteins

## Abstract

*Magnaporthe oryzae* causes rice blast disease and is responsible for major losses in rice production worldwide. Although numerous studies have focused on the interactions between *Oryza sativa* and *M. oryzae*, to date, the conserved mechanisms remain in part unclear. In this study, a comparative analysis of transcriptomes of *O. sativa* L. ssp. *japonica* cv. ‘Nipponbare’ interacting with three *M. oryzae* strains (248, 235, and 163) were performed to explore the conserved molecular mechanisms. Differentially expressed genes with similar expression patterns in the interactions between cultivar ‘Nipponbare’ and three *M. oryzae* strains were defined as Conserved Differentially Expressed Genes (CDEGs). These included 3,647 *O. sativa* CDEGs and 3,655 *M. oryzae* CDEGs. Four rice CDEGs (*LOC_Os03g19270*, *LOC_Os07g36600*, *LOC_Os05g28740*, and *LOC_Os01g32780*) encoding universal stress protein (USP) were induced within 24 h post-inoculation (hpi) by three *M. oryzae* strains. Meanwhile, overexpression of *LOC_Os07g36600* resulted in enhanced rice resistance against *M. oryzae*. Furthermore, four rice genes coding light-harvesting chlorophyll a/b-binding (LHC) protein (*LOC_Os02g52650*, *LOC_Os09g12540*, *LOC_Os11g13850*, *LOC_Os05g22730*) were also identified as CDEGs and were induced at 48 hpi, which might contribute to blast resistance through reactive oxygen species (ROS) accumulation. *MoCDIP4* is *M. oryzae* effector inducing rice cell death and were verified that include AA9 CAZy domain (namely GH61 domain). In this study, we found seven *MoCDIP4-*homologous genes coding proteins with signal peptides and AA9 CAZy domains, which were continuously up-regulated across all infection stages relative to uninoculated control. This study uncovered that genes are required for conserved mechanisms of rice-*M. oryzae* interaction, which includes rice genes encoding USP proteins and LHC proteins, as well as *M. oryzae* genes encoding AA9 proteins. This study will help us to understand how *O. sativa* responds to *M. oryzae* infections and the molecular mechanisms of *M. oryzae* pathogenicity.

## Introduction

Rice is a staple food that feeds more than half of the world’s population. Rice (*Oryzae sativa*) blast, the most destructive rice disease worldwide caused by *Magnaporthe oryzae* (a hemibiotrophic fungal pathogen), can reduce rice yield by 30% (Skamnioti and Gurr, 2009; Dean et al., 2012). Therefore, it is critical to explore the mechanism of rice-*M. oryzae* interaction and breed for rice cultivars with durable resistance to rice blast.

Approximately 24 h after attaching to the rice leaf surface, *M. oryzae* forms appressorium, which is an infectious structure that generates enormous turgor pressure and helps *M. oryzae* to penetrate into the rice cell wall (Howard and Valent, 1996; Ribot et al., 2008). Then, specialized hyphae are produced and expand within rice cells. This process is defined as the biotrophic stage, which lasts for 48 h after adhesion to the leaf surface (Wang et al., 2014). The infection then switches to the necrotrophic phase, during which the rice cells lose viability and disease lesions become evident on the leaf surface.

Plant-pathogen interactions follow the ‘zig-zag’ model, which shows that plants have evolved two main types of innate immunity: pathogen-associated molecular pattern (PAMP)-triggered immunity (PTI) and effector-triggered immunity (ETI) (Jones and Dangl, 2006). The PTI system is activated upon direct recognition of PAMPs by two major types of host pattern-recognition receptors (PRRs) (Ausubel, 2005), namely, receptor-like kinases (RLKs) and receptor-like proteins (RLPs) (Boutrot and Zipfel, 2017). The activated PTI signaling subsequently activates downstream targets that result in ROS production, stomatal closure, MAPK activation, and production of defense hormones (Yuan et al., 2021). Pathogens deliver a variety of effectors into the host cells to target PRR complex components, which inhibit the kinase activity of the PRR (Dou and Zhou, 2012). Polymorphic resistance proteins, encoded by plant resistance (*R*) genes, can directly or indirectly recognize pathogen effectors and activate ETI signaling, which results in enhanced resistance and hypersensitive response (HR) (Cui et al., 2015). Although approximately 100 rice *R* genes/alleles associated with blast resistance have been identified so far (Ashkani et al., 2015), only 37 race-specific blast *R* genes have been successfully cloned and most of them encode nucleotide-binding site-leucine-rich-repeat (NBS-LRR) proteins (Li W. et al., 2019). However, the large-scale application of rice cultivars with *R* genes is limited due to its long-term period of breeding and risk of losing resistance, which resulted from the rapid evolution of *M. oryzae* (Dean et al., 2005). Except for traditional *R* genes, defense regulator (DR) genes can regulate blast resistance and received attention due to their partial but durable broad-spectrum blast resistance (Li W. et al., 2019). Take an example, a loss-of-function allele of *Pi21*, encoding a proline-rich protein, confers broad and durable resistance against *M. oryzae* (Fukuoka et al., 2009; Liu et al., 2013). Due to conferring broad-spectrum resistance, DR genes might involve in rice response to different distinct strains of *M. oryzae*. Thus, it is important for DR genes discovery and exploration of their function through dissecting interactions between host plants and different strains of plant pathogens.

In this study, transcriptome sequencing of *O. sativa* L. ssp. *japonica* cv. ‘Nipponbare’ (Nip) inoculated with three *M. oryzae* strains (248, 235, and 162) was used to explore conserved mechanisms of rice-*M. oryzae* interaction. In our previous work, we inoculated *M. oryzae* 248, 235, and 162 on 20 rice cultivars. The pathogenicity of three *M. oryzae* strains are much different (Supplementary Table 1), which suggests *M. oryzae* 248, 235, and 162 represent different clonal lineages. Based on this, the comparative transcriptomic study was used to dissect the conserved mechanism of rice-*M. oryzae* interaction. In order to fulfill this, differentially expressed genes (DEGs) displaying similar expression patterns in the three host-pathogen interactions were defined as conserved DEGs (CDEGs). In addition to known plant defense-associated genes (i.e., *PR* genes, and diterpene biosynthesis genes), we also found that rice genes, encoding universal stress proteins (USPs) and light-harvesting chlorophyll a/b-binding protein (LHC), were induced during the infection stage. Among USP genes mentioned above, *OsUSP4* (*LOC_Os07g36600*) was found that enhance rice resistance to *M. oryzae* attack. In *M. oryzae*, seven *MoCDIP4-*homologous genes encode secreted proteins with a signal peptide and AA9 Carbohydrate-Active enzymes (CAZymes) domain, which were continuously upregulated during the whole interaction stage. Taken together, we suggest that rice USP genes, rice LHC genes, and *M. oryzae* AA9 genes involve in the conserved mechanism of rice-*M. oryzae* interaction.

## Results

### Transcriptome Sequencing and Quality Control

To study common transcriptional changes of different rice-*M. oryzae* interactions, conidia of three *M. oryzae* strains (248, 235, and 162) were used as inoculum respectively. The leaf tissues of the inoculated *japonica* cultivar ‘Nipponbare’ (Nip) were collected at 0, 8, 24, 48, 72, and 96 h post-inoculation (hpi). Nip leaves at 0 hpi and conidia of each *M. oryzae* strain were defined as control samples (CK). Total RNA was isolated from the samples described above. Low-quality bases or reads were filtered from the raw transcriptome data, and approximately 46 to 63 million pairs of reads from each sample were used in the downstream analysis. All clean reads were mapped to the reference genome of *O. sativa* L. ssp. *japonica* cv. ‘Nipponbare’ and *M. oryzae* 70-15. Approximately 20% of reads unmapped the reference genome and thus were filtered out before expression level calculation. An overview of the mapped statistics is provided in Table 1.

**TABLE 1:** Summary of alignment statistics in 15 libraries referring to *Oryza sativa* L. ssp. *japonica* genome.

	*M. oryzae* 248 vs. Nip					*M. oryzae* 235 vs. Nip					*M. oryzae* 162 vs. Nip				
Time point	8 hpi	24 hpi	48 hpi	72 hpi	96 hpi	8 hpi	24 hpi	48 hpi	72 hpi	96 hpi	8 hpi	24 hpi	48 hpi	72 hpi	96 hpi
Total raw reads	2 × 55,811,555	2 × 46,920,607	2 × 56,953,765	2 × 51,393,645	2 × 50,164,084	2 × 53,008,393	2 × 60,794,673	2 × 49,913,931	2 × 58,804,583	2 × 47,461,423	2 × 62,116,133	2 × 51,665,591	2 × 63,521,767	2 × 58,360,057	2 × 61,278,117
Mapping to Nip (%)	81.36%	80.14%	81.47%	78.2%	57.56%	82.77%	81.32%	81.75%	79.65%	62.62%	80.41%	79.53%	77.37%	76.91%	63.94%
Mapping to *M. oryzae* (%)	0.14%	0.15%	0.08%	2.06%	22.57%	0.39%	0.68%	0.25%	3.54%	20.69%	0.18%	0.24%	0.47%	5.48%	17.51%
Unmapped (%)	18.5%	19.71%	18.45%	19.74%	19.87%	16.84%	18%	17.99%	16.81%	16.69%	19.41%	20.23%	22.17%	17.61%	18.56%

### Identification of Conserved Differentially Expressed Genes Related to Rice-*M. oryzae* Interaction

Differentially expressed genes (DEGs) were identified with adjusted *p*-values of <0.01 and at least a two-fold change in the normalized (FPKM) expression values. As Supplementary Table 2 shows, 1,913 to 3,612 *O. sativa* DEGs were identified from the interaction of rice-*M. oryzae* 248, 1,267 to 4,051 *O. sativa* DEGs from the interaction of rice-*M. oryzae* 235, and 2,143 to 4,640 *O. sativa* DEGs from the interaction of rice-*M. oryzae* 162. In addition, 4,451 to 5,212, 3,982 to 4,904, and 3,623 to 4,542 DEGs were detected from *M. oryzae* 248, 235, and 162, respectively.

**FIGURE 1:**
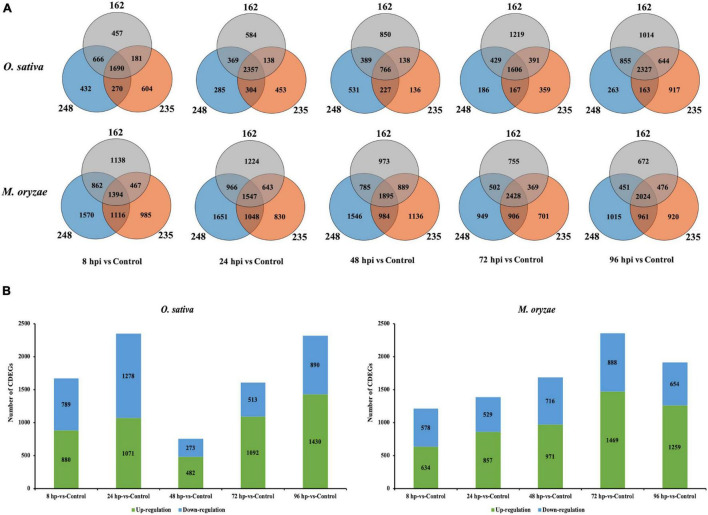
Overview of differentially expressed genes (DEGs) identified in interactions between Nipponbare (Nip) and three strains of *Magnaporthe oryzae* (248, 235, and 162) at 8, 24, 48, 72, and 96 hpi. **(A)** Venn diagram displaying overlap between DEGs in interactions between Nipponbare (Nip) and three strains of *M. oryzae*. OS represent *Oryzae sativa* L. ssp. *japonica* cv. Nipponbare and MG represent *Magnaporthe oryzae*. **(B)** Expression regulation of conserved DEGs (CDEGs).

In order to explore DEGs that may involve in the conserved mechanisms of *O. sativa-M. oryzae* interaction, we focused on the CDEGs which are DEGs that displayed similar transcriptional changes in the three interactions. As Figure 1 and Supplementary Table 3 displayed, in *O. sativa*, there were 1,690 CDEGs (880 upregulated and 789 downregulated) at 8 hpi, 2,357 CDEGs (1,071 upregulated and 1,278 downregulated) at 24 hpi, 766 CDEGs (482 upregulated and 273 downregulated) at 48 hpi, 1,606 CDEGs (1,092 upregulated and 513 downregulated) at 72 hpi, and 2,327 CDEGs (1,430 upregulated and 890 downregulated) at 96 hpi. Additionally, in *M. oryzae* there were 1,394 CDEGs (634 upregulated and 578 downregulated) at 8 hpi, 1,547 CDEGs (857 upregulated and 529 downregulated) at 24 hpi, 1,895 CDEGs (971 upregulated and 716 downregulated) at 48 hpi, 2,428 CDEGs (1,469 upregulated and 888 downregulated) at 72 hpi, and 2,024 CDEGs (1,259 upregulated and 654 downregulated) at 96 hpi. In total, 3,647 and 3,655 CDEGs were identified in *O. sativa* and *M. oryzae*, respectively.

Domain annotation of CDEGs identified at every timepoint was performed. The top10 domains of CDEGs at every timepoint were provided in Supplementary Table 4. p450 (PF00067), Pkinase (PF00069), and zf-C3HC4 (PF00097) were found across all timepoints. The CDEGs containing LRR_1 (PF00560), Pkinase_Tyr (PF07714), RRM_1 (PF00076) and Epimerase (PF01370) were specific at early infection stage (8 and 24 hpi). LRR_1 and Pkinase were defined as hallmarks of receptor-like kinases (RLKs) superfamily, which act as important players in rice defense. Take an example, Takai et al. (2008) reported OsFLS2, an RLK protein homologous to flg22, involve in flagellin perception so that promotes rice resistance. We thereby inferred the overrepresented RLKs detected at 8 and 24 hpi may relate to *M. oryzae* PAMPs perception, such as chitin. The CDEGs containing PTR2 (PF00854), WRKY (PF03106), AMP-binding (PF00501), and Aa_trans (PF01490) were specific at the late infection stage (72 and 96 hpi). Meanwhile, CDEGs specific at 48 hpi were found that contained domain of AP2 (PF00847).

### Gene Ontology Enrichment Analysis of the Conserved Differentially Expressed Genes

The results of Gene Ontology (GO) enrichment analysis of the *O. sativa* CDEGs is provided in Supplementary Table 5. Rice CDEGs, downregulated at 8, 24, 72, and 96 hpi, were enriched in the GO terms ‘photosynthesis, light harvesting in photosystem I,’ ‘photosynthetic electron transport in photosystem I,’ and ‘chlorophyll-binding.’ This indicates that these putative photosynthesis-associated CDEGs may be linked to the reduction in rice green leaf area during infection (Azizi et al., 2015; Sebela et al., 2018). Notably, several rice CDEGs that were upregulated from 48 to 96 hpi were found to be enriched in ‘cinnamic acid biosynthetic process,’ ‘cinnamic acid metabolic process,’ ‘diterpene phytoalexin biosynthetic process,’ and ‘phytoalexin biosynthetic process.’ This finding supports the fact that phytohormones, phytoalexins, and diterpene secondary metabolites play important roles in the rice defense system (Bleecker and Kende, 2000; Li N. et al., 2019). Supplementary Table 6 shows that *M. oryzae* CDEGs were upregulated from 8 to 96 hpi and enriched in the GO terms ‘carboxylic ester hydrolase activity,’ ‘carbohydrate metabolic process,’ ‘endo-1,4-beta-xylanase activity,’ and ‘endo-1,4-beta-xylanase activity,’ which implies these *M. oryzae* CDEGs may involve in the decomposition of the rice cell wall.

### Pathway Enrichment Analysis of the Conserved Differentially Expressed Genes Identified in *O. sativa* and *M. oryzae*

The CDEGs of *O. sativa* and *M. oryzae* were mapped against the Kyoto Encyclopedia of Genes and Genomes (KEGG) database (Kanehisa, 2002) and pathway enrichment analyses were performed. In *O. sativa*, we found several CDEGs at 8 to 96 hpi were enriched in photosynthesis-associated pathways, such as ‘photosynthesis - antenna proteins’ and ‘porphyrin and chlorophyll metabolism.’ (Supplementary Table 7). CDEGs at 48 to 96 hpi were enriched in ‘diterpenoid biosynthesis’ and ‘plant-pathogen interaction’ pathways that are linked to the plant defense system.

**FIGURE 2:**
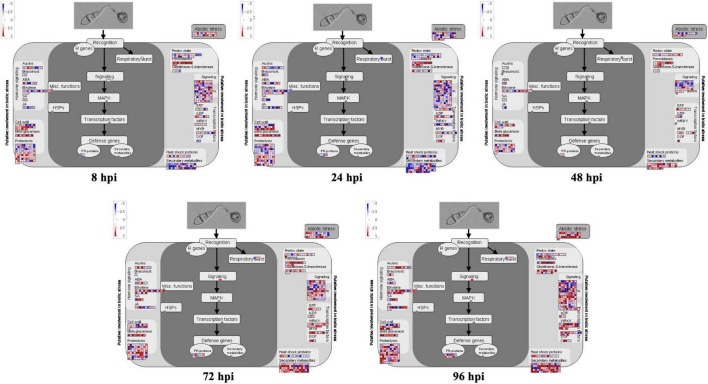
MapMan overviews of biotic stress display transcriptional change at 8, 24, 48, 72, and 96 hpi. CDEGs are significantly upregulated (red) and downregulated (blue) in inoculated leaf samples. Individual genes are represented by small squares. The scale bar displays log2-transformed fold changes.

In this study, MapMan software was used to generate overviews of the biotic stress response and metabolism in rice. We found that more rice CDEGs were upregulated during late infection stages (72 to 96 hpi) compared to early infection stages (8 to 48 hpi), and most of them were assigned terms such as ‘PR-proteins,’ ‘SA,’ ‘JA,’ ‘ethylene,’ ‘WRKY,’ ‘MYB,’ ‘secondary metabolites,’ ‘glutathione-S-transferase,’ and ‘respiratory burst’ (Figure 2). Many CDEGs, assigned to ‘cell wall’ and ‘peroxidases,’ were found to be upregulated from 8 to 24 hpi. Not surprisingly, no CDEGs were detected in ‘*R* genes.’ We also noticed that many rice CDEGs, downregulating at 8, 24, 72, and 96 hpi but upregulating at 48 hpi, were assigned to the term ‘light reactions’ (Supplementary Figure 1), which is consistent with the result of GO enrichment analysis.

For *M. oryzae*, CDEGs at all timepoints were enriched in the pathways ‘biosynthesis of antibiotics,’ ‘nitrogen metabolism,’ and ‘starch and sucrose metabolism’ (Supplementary Table 8). In addition, the pathways of ‘riboflavin metabolism,’ ‘ubiquinone and another terpenoid-quinone biosynthesis,’ ‘phenylalanine metabolism,’ and ‘carbon metabolism’ include CDEGs at early infection stages (8 to 24 hpi). CDEGs at late infection stages (72 to 96 hpi) were enriched in the ‘steroid biosynthesis,’ ‘glycerolipid metabolism,’ ‘pentose and glucuronate interconversions,’ and ‘other glycan degradation’ pathways.

### Co-expression Clustering of the Conserved Differentially Expressed Genes Identified in *O. sativa* and *M. oryzae*

**FIGURE 3:**
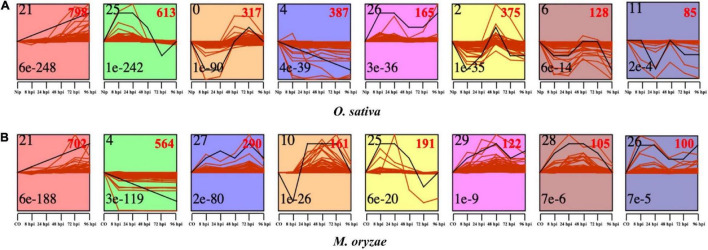
Short Time-series Expression Miner (STEM) toolkit identified the temporal expression profiles of CDEGs in *O. sativa* **(A)** and *M. oryzae* **(B)** with a *p*-value < 0.05. The number on the top left corner of each profile box was Profile ID assigned by STEM, the number on the bottom left represents the adjusted *p*-value, and the number on the top right corner represents the CDEGs count of each cluster.

In order to dissect of the expression pattern of CDEGs identified in *O. sativa* and *M. oryzae*, we performed co-expression analysis through the Short Time-series Expression Miner (STEM) toolkit (Ernst and Bar-Joseph, 2006). In brief, 2,869 of the 3,647 rice CDEGs and 2,235 of the 3,655 *M. oryzae* CDEGs were divided into eight STEM profiles with *E*-values < 0.01 respectively (Figure 3), which means that CDEGs with similar expression patterns were put into the same STEM profiles.

Rice CDEGs used for co-expression analysis were provided in Supplementary Table 9. Profiles 25 and 26 consists of CDEGs that were specifically upregulated during the early interaction stages (8 to 24 hpi); profiles 0, 2, and 6 contain CDEGs that were specifically upregulated during the late interaction stages (48 to 96 hpi); continuously upregulated rice CDEGs were put into profile 21, and CDEGs in profile 4 showed decreasing expression trends during all infection stages. The result of pathway enrichment analysis for each rice co-expression profile was provided in Supplementary Table 10. For profiles of early upregulation (profiles 25 and 26), enriched pathways mainly include ‘Carbon metabolism,’ ‘Valine, leucine and isoleucine degradation,’ ‘Propanoate metabolism,’ and so on. For profiles of late upregulation (profile 0, 2, and 6), pathways of ‘Photosynthesis - antenna proteins,’ ‘Photosynthesis’ and ‘Carbon fixation in photosynthetic organisms’ were enriched. For profiles of continuous upregulation (profile 21), pathways of ‘Biosynthesis of secondary metabolites,’ ‘Diterpenoid biosynthesis,’ and ‘Flavonoid biosynthesis’ were enriched.

For *M. oryzae*, as Supplementary Table 11 displayed, profiles 21, 27, and 29 consisted of continuously upregulated CDEGs; profiles 25 and 26 included CDEGs that were specifically upregulated during the early infection stages; profile 10 contained CDEGs specifically upregulated during the late infection stages; CDEGs in profile 4 showed continuously decreasing expression levels from 8 to 96 hpi. The result of pathway enrichment analysis for each *M. oryzae* co-expression profile was provided in Supplementary Table 12. For profiles of early upregulation (profile 25 and 26), *M. oryzae* CDEGs in these profiles mainly enriched in the pathway of ‘Biosynthesis of secondary metabolites.’ For profiles of late upregulation (profile 10), enriched pathways mainly include the pathways of ‘Metabolic pathways.’ For profiles of continuous upregulation (profile 21, 27, and 29), pathways of ‘Biosynthesis of secondary metabolites,’ ‘Starch and sucrose metabolism,’ ‘Galactose metabolism’ and ‘Glycolysis/Gluconeogenesis’ were enriched.

### Conserved Differentially Expressed Genes of *Pathogenesis-Related* Genes Are *M. oryzae*-Responsive

*Pathogenesis-related* (*PR*) genes are essential components of PAMP-triggered immunity (Duran-Flores and Heil, 2016). Here, we collected 1,074 rice *PR* genes retrieved from Zhang et al. (2016). The 112 *O. sativa PR* genes were divided into thirteen subfamilies (*PR1*, *PR2*, *PR3*, *PR4*, *PR5*, *PR6*, *PR8*, *PR9*, *PR-10*, *PR-12*, *PR-14*, *PR-15*, and *PR-16*) (Supplementary Table 13). As Supplementary Figure 2A shows, *PR* genes in profile 21 were found across diverse PR subfamilies except for PR-12. *PR* genes in profiles 25 and 26 mainly belong to *PR8* and *PR9* subfamilies, which have putative activities of chitinase and lignin-forming peroxidases (Irigoyen et al., 2020). Based on their expression profiles, nine *PR8* genes and seven *PR9* genes were specifically upregulated during the early infection stage (Supplementary Figure 2B). PR proteins in profiles 0, 2, and 6 are concentrated in the *PR-14* subfamily. Six *PR-14* genes in profiles 2 and 6 were specifically upregulated at 48, 72, and 96 hpi, which demonstrates the importance of *O. sativa PR-14* proteins during the late host-pathogen interaction stages.

### Universal Stress Proteins Might Respond to *M. oryzae* Infection

**FIGURE 4:**
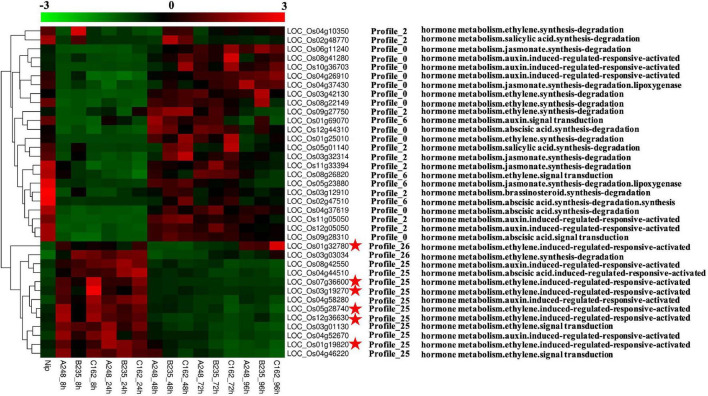
Overview of *O. sativa* CDEGs assigned to hormone metabolism or hormone signaling based on the annotation of MapMan functional categories. Heat maps display expression patterns of these *O. sativa* CDEGs. The middle part of the figure shows these *O. sativa* CDEGs belong to Profile 25, 26, 0, 2, and 6 according to co-expression clustering. The right side of the figure displays MapMan annotation of these *O. sativa* CDEGs. Among these *O. sativa* CDEGs, encoding genes of universal stress proteins (USPs), induced by ethylene according to MapMan annotation, were marked by red asterisks.

**FIGURE 5:**
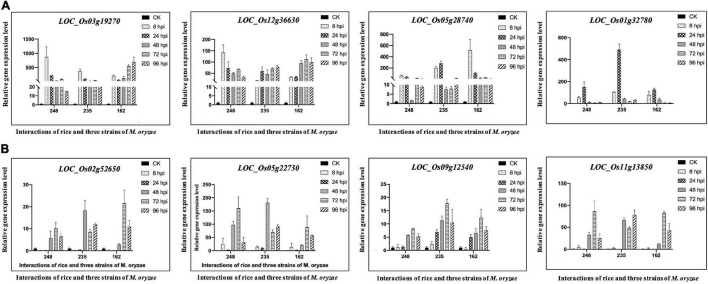
Quantitative Real-Time PCR verification of expression of selected coding genes of Light-harvesting chlorophyll a/b-binding proteins and universal stress proteins in *O. sativa* L. ssp. *japonica* cv. Nipponbare (Nip) during interactions with *Magnaporthe oryzae* 248, 235, and 162. Un-incubated Nip plant was used as the control sample and normalized to 1.0. The actin genes of Nip (*LOC_Os03g50885*) were used as internal reference genes. Data displayed as the average of three biological replicates and error bars indicate standard deviations. 8, 24, 48, 72, and 96 hpi represent hours post-incubation with three *M. oryzae* strains. **(A)** Quantitative Real-Time PCR verification for Light-harvesting chlorophyll a/b-binding proteins coding genes in Nip. **(B)** Quantitative Real-Time PCR verification for universal stress protein-coding genes in Nip.

Plant phytohormones, such as salicylic acid (SA), jasmonate acid (JA), and ethylene (ET), are essential regulators of the plant defense system, which activate the appropriate and effective responses to pathogen infection. Using MapMan analysis, we found six rice CDEGs (*LOC_Os01g32780*, *LOC_Os03g19270*, *LOC_Os05g28740*, *LOC_Os12g36630*, *LOC_Os07g36600*, and *LOC_Os01g19820*), annotated as universal stress proteins (USPs) and clustered in profiles 25 or 26 (Table 2), were assigned to the branch pathways of ‘hormone metabolism and ‘ethylene induced regulated-responsive-activated’ (BinCode 17.5.3). Figure 4 shows the six USP-coding genes were specifically upregulated from 8 to 24 hpi. the qRT-PCR result revealed that transcripts of *LOC_Os03g19270*, *LOC_Os07g36600*, *LOC_Os05g28740*, and *LOC_Os01g32780* were highly abundant during the early interaction stage (8 and 24 hpi), with almost 15 to 1200-fold compared to the control sample (Figure 5A), which suggest that *LOC_Os03g19270*, *LOC_Os07g36600*, *LOC_Os05g28740*, and *LOC_Os01g32780* might involve in the conserved mechanism of responding to *M. oryzae* attack.

**TABLE 2:** Assignment of *O. sativa* CDEGs in plant hormone pathways according to MapMan.

Category	BinCode	BinName	Gene	STEM Profile	Description
Ethylene	17.5.3	hormone metabolism.ethylene.induced-regulated-responsive-activated	LOC_Os01g32780	Profile_26	universal stress protein (USP) family protein
Ethylene	17.5.1	hormone metabolism.ethylene.synthesis-degradation	LOC_Os03g03034	Profile_26	DMR6 (Downy Mildew Resistant 6); oxidoreductase/oxidoreductase
Ethylene	17.5.3	hormone metabolism.ethylene.induced-regulated-responsive-activated	LOC_Os07g36600	Profile_25	universal stress protein (USP) family protein
Ethylene	17.5.3	hormone metabolism.ethylene.induced-regulated-responsive-activated	LOC_Os03g19270	Profile_25	universal stress protein (USP) family protein
Ethylene	17.5.3	hormone metabolism.ethylene.induced-regulated-responsive-activated	LOC_Os05g28740	Profile_25	universal stress protein (USP) family protein
Ethylene	17.5.3	hormone metabolism.ethylene.induced-regulated-responsive-activated	LOC_Os12g36630	Profile_25	universal stress protein (USP) family protein
Ethylene	17.5.2	hormone metabolism.ethylene.signal transduction	LOC_Os03g01130	Profile_25	unknown protein
Ethylene	17.5.3	hormone metabolism.ethylene.induced-regulated-responsive-activated	LOC_Os01g19820	Profile_25	universal stress protein (USP) family protein
Ethylene	17.5.2	hormone metabolism.ethylene.signal transduction	LOC_Os04g46220	Profile_25	ethylene-responsive transcription factor, putative
Auxins	17.2.3	hormone metabolism.auxin.induced-regulated-responsive-activated	LOC_Os08g42550	Profile_25	unknown protein
Auxins	17.2.3	hormone metabolism.auxin.induced-regulated-responsive-activated	LOC_Os04g58280	Profile_25	unknown protein
Auxins	17.2.3	hormone metabolism.auxin.induced-regulated-responsive-activated	LOC_Os04g52670	Profile_25	Auxin-responsive SAUR gene family member, expressed
ABA	17.1.3	hormone metabolism.abscisic acid.induced-regulated-responsive-activated	LOC_Os04g44510	Profile_25	GRAM domain-containing protein/ABA-responsive protein-related
Ethylene	17.5.1	hormone metabolism.ethylene.synthesis-degradation	LOC_Os04g10350	Profile_2	2-oxoglutarate-dependent dioxygenase, putative
Ethylene	17.5.1	hormone metabolism.ethylene.synthesis-degradation	LOC_Os03g42130	Profile_0	oxidoreductase, 2OG-Fe(II) oxygenase family protein
Ethylene	17.5.1	hormone metabolism.ethylene.synthesis-degradation	LOC_Os08g22149	Profile_0	LEJ2 (Loss of the timing Of ET and JA Biosynthesis 2)
Ethylene	17.5.1	hormone metabolism.ethylene.synthesis-degradation	LOC_Os09g27750	Profile_2	EFE (Ethylene-forming Enzyme); 1-aminocyclopropane-1-carboxylate oxidase
Ethylene	17.5.1	hormone metabolism.ethylene.synthesis-degradation	LOC_Os01g25010	Profile_0	oxidoreductase, 2OG-Fe(II) oxygenase family protein
Ethylene	17.5.2	hormone metabolism.ethylene.signal transduction	LOC_Os11g33394	Profile_0	unknown protein
Ethylene	17.5.2	hormone metabolism.ethylene.signal transduction	LOC_Os08g26820	Profile_6	unknown protein
Auxins	17.2.3	hormone metabolism.auxin.induced-regulated-responsive-activated	LOC_Os08g41280	Profile_0	auxin-responsive family protein
Auxins	17.2.3	hormone metabolism.auxin.induced-regulated-responsive-activated	LOC_Os10g36703	Profile_0	auxin-responsive family protein
Auxins	17.2.3	hormone metabolism.auxin.induced-regulated-responsive-activated	LOC_Os04g26910	Profile_0	ATB2; oxidoreductase
Auxins	17.2.2	hormone metabolism.auxin.signal transduction	LOC_Os01g69070	Profile_6	PIN5 (PIN-FORMED 5); auxin:hydrogen symporter/transporter
Auxins	17.2.3	hormone metabolism.auxin.induced-regulated-responsive-activated	LOC_Os11g05050	Profile_2	AILP1
Auxins	17.2.3	hormone metabolism.auxin.induced-regulated-responsive-activated	LOC_Os12g05050	Profile_0	AILP1
JA	17.7.1.5	hormone metabolism.jasmonate.synthesis-degradation	LOC_Os06g11240	Profile_0	OPR2
JA	17.7.1.2	hormone metabolism.jasmonate.synthesis-degradation.lipoxygenase	LOC_Os04g37430	Profile_0	LOX3; electron carrier/lipoxygenase/metal ion binding/oxidoreductase
ABA	17.1.1.1.10	hormone metabolism.abscisic acid.synthesis-degradation	LOC_Os12g44310	Profile_0	CCD1 (Carotenoid Cleavage Dioxygenase 1); 9-cis-epoxycarotenoid dioxygenase
ABA	17.1.1.1.1	hormone metabolism.abscisic acid.synthesis-degradation	LOC_Os04g37619	Profile_0	ABA1 (ABA deficient 1); zeaxanthin epoxidase
ABA	17.1.2	hormone metabolism.abscisic acid.signal transduction	LOC_Os09g28310	Profile_0	ABF4 (ABRE binding factor 4); DNA/protein binding
SA	17.8.1	hormone metabolism.salicylic acid.synthesis-degradation	LOC_Os02g48770	Profile_2	BSMT1; S-adenosylmethionine-dependent methyltransferase
SA	17.8.1	hormone metabolism.salicylic acid.synthesis-degradation	LOC_Os05g01140	Profile_2	S-adenosyl-L-methionine:carboxyl methyltransferase family protein
JA	17.7.1.4	hormone metabolism.jasmonate.synthesis-degradation	LOC_Os03g32314	Profile_2	AOC3 (ALLENE OXIDE CYCLASE 3); allene-oxide cyclase
Brassinost	17.3.1.2.99	hormone metabolism.brassinosteroid.synthesis-degradation	LOC_Os03g12910	Profile_2	XF1; squalene monooxygenase
JA	17.7.1.2	hormone metabolism.jasmonate.synthesis-degradation.lipoxygenase	LOC_Os05g23880	Profile_6	LOX5; electron carrier/lipoxygenase/metal ion binding/oxidoreductase
ABA	17.1.1.1.10	hormone metabolism.abscisic acid.synthesis-degradation.synthesis	LOC_Os02g47510	Profile_6	NCED4 (Nine-cis-epoxycarotenoid Dioxygenase 4)

### *OsUSP4* (*LOC_Os07g36600*) Is Involved in Blast Resistance

**FIGURE 6:**
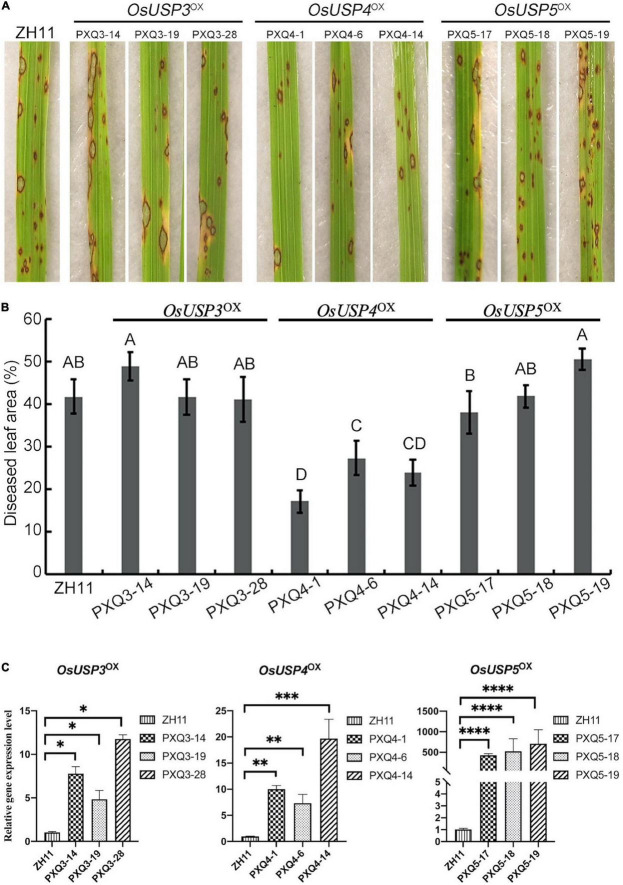
Disease reactions of ZH11, *OsUSP3*^OX^, *OsUSP4*^OX^, *OsUSP5*^OX^ leaves incubated by Guy11. **(A)** Photographs showing disease reaction of indicated rice lines and races: wild-type (ZH11); overexpression transgenic line of *OsUSP3*^OX^ (PXQ3-14, PXQ3-19, PXQ3-28), *OsUSP4*^OX^ (PXQ4-1, PXQ4-6, and PXQ4-14), and *OsUSP5*^OX^ (PXQ5-17, PXQ5-18, PXQ5-19). **(B)** Disease lesion area was assessed by Image J. Lesions were photographed and measured or scored at 6 days post-inoculation by isolation of Guy11. A, B, C, and D represent the significant difference (one-way ANOVA test, *P* < 0.01). **(C)** qRT-PCR was used for expression-level evaluation of universal stress proteins (USPs) coding genes in ZH11 and corresponding transgenic lines: *OsUSP3* in PXQ3-14, PXQ3-19, and PXQ3-28; *OsUSP4* in PXQ4-1, PXQ4-6 and PXQ4-14; *OsUSP5* in PXQ5-17, PXQ5-18, and PXQ5-19. *T*-test Asterisks denote significant differences compared to ZH11 plants (Student *t*-test with two-sided and three replicates, *P < 0.05; ^**^P < 0.01; ^***^P < 0.001; ^****^P < 0.0001).

According to the analysis above, we found four USPs may involve in the conserved mechanism of response to *M. oryzae*. To investigate this, overexpression transgenic line of three rice USPs coding genes (*OsUSP3: LOC_Os03g19270*; *OsUSP4*: *LOC_Os07g36600*; *OsUSP5*: *LOC_Os05g28740*) were produced via PXQ vector by transforming *PXQ:OsUSPs* into ZH11 cultivar (wild type, Zhonghua 11), and were designed as *OsUSP3*^OX^, *OsUSP4*^OX^, and *OsUSP5*^OX^ respectively. A total of seventy-six independent transgenic T_1_ lines were obtained (thirty-two for *OsUSP3*^OX^, fourteen for *OsUSP4*^OX^, thirty for *OsUSP5*^OX^). Based on the qRT-PCR analysis, it is notable that *OsUSP3*, *OsUSP4*, and *OsUSP5* showed significantly higher expression levels compare to that in ZH11 (Figure 6C). Thus, overexpression transgenic lines of *OsUSP3*^OX^ (PXQ3-14, PXQ3-19, PXQ3-28), *OsUSP4*^OX^ (PXQ4-1, PXQ4-6, PXQ4-14), and *OsUSP5*^OX^ (PXQ5-17, PXQ5-18, PXQ5-19, 5-25) were selected for inoculation assays. Selected overexpression transgenic lines were grown in a greenhouse for 2 weeks and were inoculated with *M. oryzae* isolates Guy11. Seven-days after inoculation, the diseased leaf area of PXQ4-1, PXQ4-6 and PXQ4-14 are significantly smaller than that of ZH11 (Figures 6A,B). However, there were no significant differences between the lesion area of ZH11 and overexpression transgenic lines of *OsUSP3*^OX^ and *OsUSP5*^OX^. Moreover, ZH11 and three overexpression transgenic lines of *OsUSP4* were also inoculated by *M. oryzae* 248, 235, and 162. As Supplementary Figure 3 showed, the diseased leaf ZH11 were 21.11, 25.52, and 12.32% after being inoculated by *M. oryzae* 248, 235, and 162, which are significantly larger than that of three overexpression transgenic lines of *OsUSP4*. This result suggests that *OsUSP4* (*LOC_Os07g36600*) might be blast pathogen-responsive and slow disease response to rice blast.

### Light-Harvesting Chlorophyll a/b-Binding Protein Is Associated With Response to *M. oryzae*

Based on previous KEGG and MapMan analysis, we found that photosynthesis-associated pathways are important during the rice-*M. oryzae* interaction and rice genes involved in these pathways encode light-harvesting chlorophyll a/b-binding protein (LHC). LHC superfamily consists of eight subfamilies: *Lhca*, *Lhcb*, *PsbS* (photosystem II subunit S), *FCII* (ferrochelatase II), *OHP* (one-helix protein), *SEP* (stress-enhanced protein), *ELIP* (early light-induced protein), and *Psb33* (photosystem II protein 33) (Klimmek et al., 2006; Engelken et al., 2010; Zou and Yang, 2019).

**FIGURE 7:**
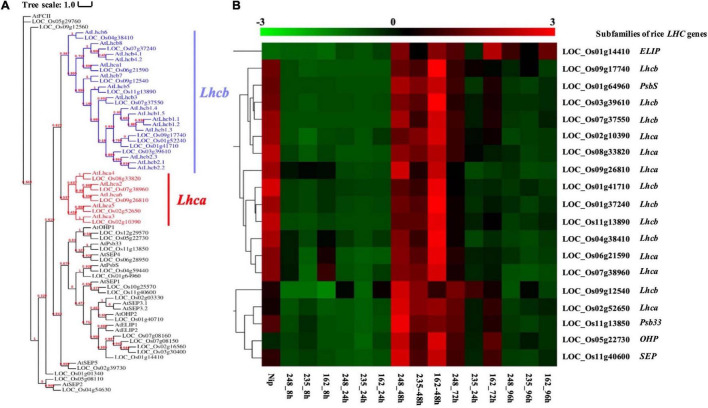
Analysis of *O. sativa* light-harvesting chlorophyll a/b-binding proteins (LHCs) superfamily. **(A)** Phylogenetic tree of LHCs proteins identified in *O. sativa* and *Arabidopsis thaliana* Maximum likelihood tree, with 1,000 bootstraps (values displayed per branch). Subfamilies of *Lhcb* and *Lhca* were marked in blue and red color. **(B)** RNAseq Expression level of *Lhcb* and *Lhca* genes during interacting with *M. oryzae*. The LHCs subfamily and their homologous genes in *Arabidopsis thaliana* were listed on the right side of the figure.

We collected known *LHC* genes from Umate (2010) and obtained 34 rice *LHC* genes following the method of Zhao et al. (2020) (Figure 7A). Among them, 19 rice *LHC* genes were identified as rice CDEGs and most of them were arranged into STEM Profile_2, which include six *Lhca* genes, eight *Lhcb* genes, one *PsbS* gene (*LOC_Os01g64960*), one *OHP* gene (*LOC_Os05g22730*), one *SEP* gene (*LOC_Os11g40600*) and one *Psb33* gene (*LOC_Os01g64960*) (Table 3). Combined with the expression profile, it is obvious that these rice *LHC* genes were induced at 48 hpi (Figure 7B), which include *LHCB5* (*LOC_Os11g13890*). Liu et al. (2019) reported *LHCB5* phosphorylation was activated by *M. oryzae* Guy11 and contributes to blast resistance through ROS accumulation. Through verification of qRT-PCR assays, transcription of *LOC_Os02g52650* (*Lhca* subfamily), *LOC_Os09g12540* (*Lhcb* subfamily), *LOC_Os11g13850* (*Psb33* subfamily), and *LOC_Os05g22730* (*OHP* subfamily) obviously increased with 5- to 150-fold expression level since 48 hpi (Figure 5B). Taken together, we speculate that *LOC_Os02g52650*, *LOC_Os09g12540*, *LOC_Os11g13850*, and *LOC_Os05g22730* might involve blast resistance with a similar function to *LHCB5*.

**TABLE 3:** Detailed information of LHC superfamily genes in *O. sativa*.

	Gene	Ortholog in *Arabidopsis*		Pfam Annotation		STEM Profile	Chloroplast transit peptide
*Lhca*	LOC_Os06g21590	*AtLhca1*	AT3G54890	Chloroa_b-bind	PF00504	Profile_2	Y
	LOC_Os07g38960	*AtLhca2*	AT3G61470	Chloroa_b-bind	PF00504	Profile_2	Y
	LOC_Os02g10390	*AtLhca3*	AT1G61520	Chloroa_b-bind	PF00504	Profile_2	Y
	LOC_Os08g33820	*AtLhca4*	AT3G47470	Chloroa_b-bind	PF00504	Profile_2	Y
	LOC_Os02g52650	*AtLhca5*	AT1G45474	Chloroa_b-bind	PF00504	Profile_2	Y
	LOC_Os09g26810	*AtLhca6*	AT1G19150	Chloroa_b-bind	PF00504	Profile_2	Y
*Lhcb*	LOC_Os01g41710	*AtLhcb1.5*	AT2G34420	Chloroa_b-bind	PF00504	Profile_2	Y
	LOC_Os01g52240	*AtLhcb1.5*	AT2G34420	Chloroa_b-bind	PF00504	−	Y
	LOC_Os09g17740	*AtLhcb1.5*	AT2G34420	Chloroa_b-bind	PF00504	Profile_2	Y
	LOC_Os03g39610	*AtLhcb2.1*	AT2G05100	Chloroa_b-bind	PF00504	Profile_2	−
	LOC_Os07g37550	*AtLhcb3*	AT5G54270	Chloroa_b-bind	PF00504	Profile_2	−
	LOC_Os07g37240	*AtLhcb4.2*	AT3G08940	Chloroa_b-bind	PF00504	Profile_2	Y
	LOC_Os11g13890	*AtLhcb5*	AT4G10340	Chloroa_b-bind	PF00504	Profile_2	Y
	LOC_Os04g38410	*AtLhcb6*	AT1G15820	Chloroa_b-bind	PF00504	Profile_2	Y
	LOC_Os09g12540	*AtLhcb7*	AT1G76570	Chloroa_b-bind	PF00504	Profile_2	Y
*PsbS*	LOC_Os01g64960	*AtPsbS*	AT1G44575	Chloroa_b-bind	PF00504	Profile_2	Y
	LOC_Os04g59440	*AtPsbS*	AT1G44575	Chloroa_b-bind	PF00504	−	Y
*ELIP*	LOC_Os07g08150	*AtELIP1*	AT3G22840	Chloroa_b-bind	PF00504	−	Y
	LOC_Os07g08160	*AtELIP1*	AT3G22840	Chloroa_b-bind	PF00504	−	Y
	LOC_Os01g14410	*AtELIP2*	AT4G14690	Chloroa_b-bind	PF00504	Profile_21	Y
	LOC_Os02g16560	*AtELIP1*	AT3G22840	Chloroa_b-bind	PF00504	−	Y
	LOC_Os03g30400	*AtELIP1*	AT3G22840	−	−	−	Y
*OHP*	LOC_Os05g22730	*AtOHP1*	AT5G02120	−	−	Profile_2	Y
	LOC_Os12g29570	*AtOHP1*	AT5G02120	−	−	−	Y
	LOC_Os01g40710	*AtOHP2*	AT1G34000	−	−	−	Y
*SEP*	LOC_Os10g25570	*AtSEP1*	AT4G34190	−	−	−	Y
	LOC_Os11g40600	*AtSEP1*	AT4G34190	−	−	Profile_2	Y
	LOC_Os04g54630	*AtSEP2*	AT2G21970	Chloroa_b-bind	PF00504	−	−
	LOC_Os02g03330	*AtSEP3.1*	AT4G17600	−	−	−	Y
	LOC_Os06g28950	*AtSEP4*	AT3G12345	−	−	−	Y
	LOC_Os02g39730	*AtSEP5*	AT4G28025	−	−	−	Y
*Psb33*	LOC_Os11g13850	*AtPsb33*	AT1G71500	−	−	Profile_2	Y
*FCII*	LOC_Os05g29760	*AtFCII*	AT2G30390	Ferrochelatase	PF00762	−	Y
	LOC_Os09g12560	*AtFCII*	AT2G30390	Ferrochelatase	PF00762	−	−

### Diterpene Phytoalexins Are Related to the Conserved Mechanism of the Rice Response to *M. oryzae* Infection

Some continuously upregulated rice CDEGs were found to be enriched in secondary metabolite biosynthesis pathways according to the MapMan analysis. Thus, antiSMASH^1^ was used to predict secondary metabolite biosynthesis clusters in rice using the default parameters.

A total of 40 *O. sativa* secondary metabolite biosynthesis clusters were identified (Supplementary Figure 4A), which consist of 14 saccharide clusters, six lignan clusters, five polyketide clusters, and five terpene clusters. Fifteen rice CDEGs were identified as being associated with terpene biosynthesis, which obviously exceeds the number of CDEGs in other secondary metabolite clusters (Supplementary Table 14). These terpene biosynthesis-associated CDEGs were distributed in clusters c12 and c16 (Supplementary Table 14). Five of these CDEGs (i.e., *LOC_Os02g36020*, *LOC_Os02g36140*, *LOC_Os02g36210*, *LOC_Os04g09900*, and *LOC_Os04g10060*) mapped to the ‘diterpenoid biosynthesis’ pathway and encode core terpene biosynthesis enzymes (Supplementary Figures 4B,C). Notably, the four CDEGs coding core terpene biosynthesis enzymes were arranged into profile 21 (Supplementary Table 14), which suggests continuously increasing their expression level during whole rice-*M. oryzae* interaction stage. Furthermore, the activity prediction of the four core terpene biosynthesis enzymes was also performed. *LOC_Os02g36210* encode ent-copalyl diphosphate synthase (EC: 5.5.1.13). *LOC_Os02g36140* are predicted to function as ent-cassa-12,15-diene synthase (EC: 4.2.3.28). *LOC_Os04g09900* have an enzymatic activity as syn-copalyl-diphosphate synthase (EC: 5.5.1.14). *LOC_Os04g10060* was annotated as syn-pimara-7,15-diene synthase (EC: 4.2.3.35).

### Alternative Splicing of *O. sativa* RCD1-SRO-TAF4 (RST) Gene Associated With Response to *M. oryzae*

Putative alternative splicing (AS) events that occurred in CDEGs of *O. sativa* and *M. oryzae* were identified *via* rMATs software (false discovery rate (FDR) cutoff of ≤0.05 and ΔPSI of ≥10%). There are no AS events predicted among *M. oryzae* CDEGs. In contrast, a total of 24 AS events, distributed across 15 *O. sativa* CDEGs, were detected (Table 4). Thirteen AS events were intron retention (IR), which was followed by alternative 3′ sites (A3SS, three AS events), alternative 5′ sites (A5SS, three events), exon-skipping (SE, three events), and mutually exclusive exons (MXE, two events). Overall, almost all AS events occurred at 8 or 24 hpi of rice-*M. oryzae* interaction. These AS events-occurred *O. sativa* CDEGs encode proteins containing diverse domains such as Pkinase (PF00069), 14_3_3 (PF00244), TPMT (PF05724), zf-U1 (PF06220), Homeobox (PF00046), adh_short (PF00106), and RST (PF12174). Seven of these CDEGs were annotated as “unknown function.” Interestingly, *LOC_Os10g42710* (*OsSRO1a*), with an RST domain, was found to suppress *Xanthomonas oryzae* pv *oryzae* (*Xoo*) infection through the *OsMYC2*-mediated JA signaling pathway (Kashihara et al., 2020). There are seven transcripts of *LOC_Os10g42710* (*LOC_Os10g42710.1* to *LOC_Os10g42710.7*) that were detected based on *in silico* prediction (Supplementary Figure 5A). *LOC_Os10g42710.2* contain complete RST domain and are major transcripts with the highest expression value at 8 hpi of rice respond to three *M. oryzae* strains (Supplementary Figure 5B). An intron retention event occurred at 8 and 24 hpi, which result in the gene model of *LOC_Os10g42710* being switched from other transcripts to *LOC_Os10g42710.2*. Taken together, *LOC_Os10g42710.2*, containing complete RST domain, might be associated with regulation of JA-induced resistance compared to other transcripts.

**TABLE 4:** Annotation of 15 *O. sativa* CDEGs with 24 AS events.

Species	GeneID	AS Type	Infection stage	STEM Profile	Pfam domain	Pfam ID
*O. Sativa*	LOC_Os01g19150	IR/IR	8 hpi/24 hpi	21	Pkinase	PF00069
*O. Sativa*	LOC_Os02g37834	MXE/MXE	8 hpi/24 hpi	23	NA	NA
*O. Sativa*	LOC_Os03g12064	A5SS	8 hpi	21	NA	NA
*O. Sativa*	LOC_Os04g38870	IR/IR	8 hpi/24 hpi	21	14_3_3	PF00244
*O. Sativa*	LOC_Os06g06040	SE/SE	8 hpi/24 hpi	23	TPMT	PF05724
*O. Sativa*	LOC_Os06g11170	IR	8 hpi	25	zf-U1	PF06220
*O. Sativa*	LOC_Os06g38320	IR	24 hpi	20	DUF1475	PF07343
*O. Sativa*	LOC_Os06g43860	IR	8 hpi	12	Homeobox	PF00046
*O. Sativa*	LOC_Os08g04450	A3SS	8 hpi	12	NA	NA
*O. Sativa*	LOC_Os09g25934	IR	8 hpi	23	NA	NA
*O. Sativa*	LOC_Os10g15310	SE	96 hpi	21	NA	NA
*O. Sativa*	LOC_Os10g40030	IR	48 hpi	0	adh_short	PF00106
*O. Sativa*	LOC_Os10g42710	IR/A3SS/IR/A3SS	8 hpi/8 hpi/24 hpi/24 hpi	25	RST	PF12174
*O. Sativa*	LOC_Os11g14544	A5SS/A5SS	8 hpi/24 hpi	23	NA	NA
*O. Sativa*	LOC_Os12g20390	IR/IR	8 hpi/24 hpi	21	NA	NA

### *Magnaporthe oryzae* Effectors Related to the Conserved Infection Mechanism

**FIGURE 8:**
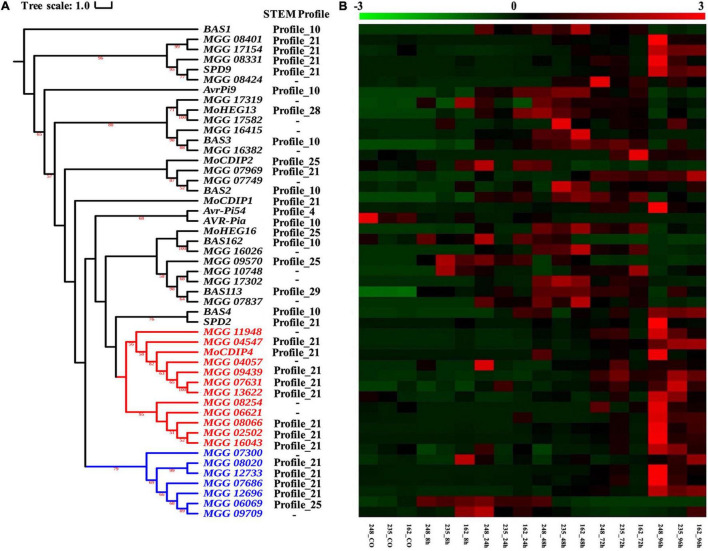
Analysis of verified and potential effectors. **(A)** Phylogenetic tree of 45 verified effectors in *M. oryzae* and their homology genes. The maximum likelihood tree with 1,000 bootstraps was displayed (values displayed per branch). Red and blue branches represent the *MoCDIP4* effector and its homolog genes. **(B)** Expression pattern of verified and potential effectors which followed the topologic order of phylogeny tree.

During the period of host colonization, *M. oryzae* secretes a set of effectors that disturb plant immune systems (van der Does and Rep, 2007; Oliva et al., 2010). In this study, 46 known *M. oryzae* effectors were retrieved from the study of Gómez Luciano et al. (2019), and 16 of these verified effectors were assigned into six STEM profiles based on the co-expression analysis (Supplementary Table 15). As Figure 8A showed, most known effector genes grouped in profile 10 (i.e., *AvrPi9*, *BAS162*, *BAS3*, *BAS1*, *BAS2*, *BAS4*, and *AVR-Pia*), which was specifically upregulated from 24 to 72 hpi (Figure 8B). *SPD9*, *MoCDIP4*, *SPD2*, and *MoCDIP1* were upregulated throughout the whole infection process and were therefore classified as profile 21. Profile 25 includes *MoCDIP2* and *MoHEG16*, which supports the idea that *MoCDIP2* and *MoHEG16* may play roles during early infection stages (from 8 to 24 hpi). Moreover, *Avr-Pi54*, *MoHEG13*, and *BAS113* belong to profiles 4, 28, and 29, respectively.

For searching more putative effector genes in the CDEGs, the BLASTP toolkit (*E*-value threshold: 1E-15) and MCL software (inflation threshold: 4.0) were used to cluster *M. oryzae* genes based on their protein sequences. As Supplementary Table 15 showed, *SPD9*, *MoCDIP2*, *MoCDIP4*, *BAS113*, *BAS162*, *MoHEG13*, *BAS3*, *BAS2*, and *BAS4* were found to have between 1 and 18 homologous genes. There are 18 homologous genes of effector *MoCDIP4* (*MGG_08409*), which is more than that of other effectors. 11 out of *MoCDIP4-*homologous genes were assigned to profile 21 (as does *MoCDIP4* itself), which showed continuous upregulation during the whole interaction stage between and three strains of *M. oryzae* (Figures 3B, 8B). Figure 8A displayed *MoCDIP4* and its homologous genes are concentrated in two distantly related clades, which suggests different functions. *MGG_11948*, *MGG_04547*, *MGG_04057*, *MGG_09439*, *MGG_07631*, *MGG_13622, MGG_08245*, *MGG_06621*, *MGG_08066*, *MGG_02502* and *MGG_16043* share same phylogenic clade and STEM profile with *MoCDIP4* (red branches in Figure 8A), which infers their similar function to *MoCDIP4*. *MoCDIP4-*homologous genes in distantly related clade (*MGG_07300*, *MGG_08020*, *MGG_12733*, *MGG_07686*, *MGG_12696*, *MGG_06069*, and *MGG_09709*) were marked by blue color in Figure 8A and displayed their continuous upregulation expression level and potential different function with *MoCDIP4*.

### Carbohydrate-Active Enzymes Associated With Pathogenicity

Phytopathogenic fungi are known to produce cell wall degrading enzymes (CWDEs) to breach the plant cell wall, which is the most important physical barrier during plant-pathogen interactions. Therefore, we predicted CAZymes in *M. oryzae* using the dbCAN web server^2^ (Yin et al., 2012), HMMER (Finn et al., 2011), DIAMOND (Buchfink et al., 2015), and Hotpep (Busk et al., 2017). We identified 399 CAZymes-coding genes and 164 of them were identified as CDEGs. Most of the CDEGs related to CAZymes belonged to the subfamilies of AA9, GH3, GH10, GH2, and GH31 (Supplementary Figure 6A and Table 5). Based on signal peptide prediction, we found ten secreted AA9 proteins (Supplementary Figure 6B), which include *MGG_04547*, *MGG_02502*, *MGG_07575*, *MGG_07686*, *MGG_12696*, *MGG_13241*, *MGG_13622*, *MGG_07631 MoCDIP4* (*MGG_08409*) and *MoAa1* (*MGG_06069*). Except for *MGG_13241* and *MGG_07575*, seven of them are homologous to *MoCDIP4* (the sequence similarities between them and *MoCDIP4* are range from 29.2 to 42.4%) (Supplementary Figure 6C). Taken together, the AA9 CAZy subfamily may play an important role in conserved mechanisms during *M. oryzae* attacking rice.

**TABLE 5:** *M. oryzae* AA9 genes in CDEGs list.

Gene ID	HMMER	STEM Profile	Signal peptide	Transmembrane	Cellular location
MGG_04547	AA9 (11–209)	Profile_21	Y (1–21)	N	Secreted
MGG_08020	AA9 (38–252)	Profile_21	N	N	Cytoplasm
MGG_12733	AA9 (39–230)	Profile_21	N	N	Cytoplasm
MGG_02502	AA9 (5–213)	Profile_21	Y (1–17)	N	Secreted
MGG_07575	AA9 (5–242)	Profile_21	Y (1–18)	N	Secreted
MGG_05364	AA9 (61–283)	Profile_21	N	N	Cytoplasm
MGG_07686	AA9 (6–227)	Profile_21	Y (1–17)	N	Secreted
MGG_08409 (*MoCDIP4*)	AA9 (7–218)	Profile_21	Y (1–19)	N	Secreted
MGG_12696	AA9 (7–221)	Profile_21	Y (1–21)	N	Secreted
MGG_13241	AA9 (7–230)	Profile_21	Y (1–19)	N	Secreted
MGG_08066	AA9 (8–206)	Profile_21	N	N	Cytoplasm
MGG_13622	AA9 (8–225)	Profile_21	Y (1–19)	N	Secreted
MGG_07631	AA9 (9–226)	Profile_21	Y (1–21)	N	Secreted
MGG_06069 (*MoAa1*)	AA9 (9–229)	Profile_25	Y (1–20)	N	Secreted

## Discussion

The *O. sativa-M. oryzae* interactions are critically important due to the huge threat *M. oryzae* infection poses to rice yield. However, the conserved molecular mechanisms underlying the *O. sativa-M. oryzae* interactions are unclear. To describe this, we compared transcriptome data from *O. sativa* L. ssp. *japonica* cv. ‘Nipponbare’ interacted with three *M. oryzae* strains (248, 235, and 162).

In conclusion, we have identified the potential roles played by members of several rice gene families in host resistance to *M. oryzae.* For example, the PR-8 and PR-9 genes were specifically upregulated during the initial infection stages, but PR-14 genes showed the opposite expression trend. Although the function of *PR* genes in blast resistance was well-known, we revealed that *PR8*, *PR9*, and *PR-14* subfamilies are an important component of conserved mechanisms of the rice response to *M. oryzae* infection. Moreover, the expression pattern of core terpene biosynthesis enzymes revealed that increasing production plays a certain role in the conserved mechanism of rice-*M. oryzae* interaction, which consists of previous publications. Shimura et al. (2007) reported that *LOC_Os02g36210* (*OsCyc2*) and *LOC_Os02g36140* (*OsDTC1*) encode major enzymes in the biosynthesis of the diterpene phytoalexins phytocassane A-E, and *LOC_Os04g09900* (*OsCyc1*) and *LOC_Os04g10060* (*OsKS4*) are responsible for the production of Momilactones A and B. In addition, regarding cytochrome P450, the expression of genes, such as *LOC_Os04g10160* (*CYP99A2*) and *LOC_Os04g09920* (*CYP99A3*), is induced by the chitin oligosaccharide elicitor of pathogens (Shimura et al., 2007).

Although the pathways of SA and JA are essential for rice resistance, we noticed the ethylene biosynthesis pathway also might play an important role in the conserved mechanism of rice-*M. oryzae* interaction. Ethylene biosynthesis is induced in response to abiotic and biotic stresses (Bleecker and Kende, 2000; Li N. et al., 2019). For example, Sauter et al. (2002) found that ethylene can regulate the expression of *OsUSP1* (one rice USP gene) during adaptation to submergence stress. This suggests that some USP genes are induced by ethylene and are associated with tolerance to several abiotic stresses, such as drought and cold (Loukehaich et al., 2012; Melencion et al., 2017). Furthermore, ERF-like transcription factors, the components of the ethylene pathway, were reported that activate the expression of genes involved in various aspects of the systemic induced defense responses (Broekaert et al., 2006). Remarkably, in this study, we found four rice USP genes (*LOC_Os03g19270*, *LOC_Os07g36600*, *LOC_Os05g28740*, and *LOC_Os01g32780*) are associated with a pathway of ‘ethylene induced regulated-responsive-activated’ and specifically upregulated at 8 and 24 hpi by three strains *M. oryzae*. We therefore speculate the four rice USPs genes might be induced by ethylene. We next obtained overexpression transgenic rice line of three USP genes above (*OsUSP3*: *LOC_Os03g19270*; *OsUSP4*: *LOC_Os07g36600*; *OsUSP5*: *LOC_Os01g32780*). The transgenic line of *OsUSP4* was found to have a milder disease progression compared to ZH11 (wild type) despite there being no difference in inoculation phenotype between ZH11 and the transgenic lines of *OsUSP3* and *OsUSP5* (Figure 6). Gou et al. (2020) recently found that *MfUSP1*, one of the USP genes in *Medicago falcata*, regulates the antioxidant defense system to maintain ROS homeostasis, which limits the growth of plant pathogens. Combined with this, we thereby suggest that induction of *OsUSP4* (*LOC_Os07g36600*) may involve the conserved mechanism of response to *M. oryzae* infection and confer partial blast resistance.

Due to the massive amount of energy required during the induction of the plant defense system (Swarbrick et al., 2006), there is an increased demand for photosynthesis, the major pathway that provides required carbon sources during plant-pathogen interactions. However, photosynthesis-related genes receive little attention in the previous rice-*M. oryzae* transcriptome studies. Herein, we noticed that the *Lha* and *Lhb* genes, two subfamilies that encode light-harvesting chlorophyll a/b-binding proteins (LHCs), were downregulated during early infection stages, which was not the expected result. A similar phenomenon was also observed by Barriuso et al. (2008) and Ishiga et al. (2009). One possible explanation that has been proposed is that the reduced photosynthesis limits carbon source availability, which can be obtained by pathogens, or that downregulating photosynthesis can protect the plant cell against oxidative damage (Blokhina et al., 2003; Bolton, 2009). In our study, we found that four photosynthesis-associated genes (*LOC_Os02g52650*: *Lhca* subfamily; *LOC_Os09g12540*: *Lhcb* subfamily; *LOC_Os11g13850*: *Psb33* subfamily; *LOC_Os05g22730*: *OHP* subfamily) were induced after 48 hpi. Combined with previous research (Liu et al., 2019), the hypothesis was proposed that the four photosynthesis-related genes in rice might promote ROS generation so that contribute to rice blast resistance, which is activated by infection of three strains *M. oryzae*. However, the detailed mechanism of this will be focused on in future studies.

The study of Chen et al. (2013) initially reported *MoCDIP4*, containing AA9 domain (namely GH61 domain), that induces light-dependent cell death in *Nicotiana benthamiana* and light-independent cell death in rice, which may facilitate the colonization of *M. oryzae*. This suggests that light harvesting in plants may be associated with *MoCDIP4*-induced cell death and even disease progression of *M. oryzae*. Here we found that 11 *MoCDIP4*-homologous genes showed a trend of continuously upregulated expression following inoculation with *M. oryzae* (Figure 8) and seven of them contain a signal peptide and AA9 CAZyme domain (Supplementary Figure 6). Among them, *MGG_13622, MGG_07631, MGG_04547*, and *MGG_02502* share the same phylogenic clade and own higher sequence similarity with *MoCDIP4*, which suggest their similar function to *MoCDIP4*. However, *MGG_07686, MGG_12696*, and *MGG_06069* locate in different phylogenic clades with *MoCDIP4* and own lower sequence similarities with *MoCDIP4*, which suggests their potential different function with *MoCDIP4*. Notably, *MoAa91* (*MGG_06069*) was found to promote appressorium formation and suppress the chitin-induced plant immune response by competing with the immune receptor chitin elicitor-binding protein precursor (*CEBiP*) (Li et al., 2020). Taken together, the induction of *MoCDIP4*-homologous genes revealed the important role of the AA9 subfamily in rice blast pathogenicity.

Overall, this study will contribute to our understanding of the conserved molecular mechanisms of rice-*M. oryzae* host-pathogen interaction. Rice USP genes, rice LHC genes, and *M. oryzae* AA9 genes might be required for the conserved mechanism of rice-*M. oryzae* interaction. We also verified that *OsUSP4* (*LOC_Os07g36600*) involve in rice resistance to *M. oryzae* attack. This study will deepen our understanding of rice-*M. oryzae* and broad ideas for further studies.

## Materials and Methods

### Plant Materials, Fungal Materials, and Growth Conditions

Wild-type (*O. sativa* L. ssp. *japonica* cv. Nipponbare (Nip) and Zhonghua 11) and overexpression transgenic lines of rice USPs coding genes (*OsUSP3*^OX^, *OsUSP4*^OX^, *OsUSP5*^OX^) were used in this study. All rice seeds were rinsed twice with demineralized water and germinated for 3 days at 28°C on sterilized wet filter paper. Germinated seeds were placed in a disposable plastic cup and grown in a greenhouse for 2 weeks (16/8 h light/dark cycle, 28 ± 2°C, and 75% humidity). Three *M. oryzae* strains of 248, 235, and 162 were selected from strains that we collected from the disease nursery of two municipal rice breeding institutes (Jintan: 31°40′20″N, 119°21′34″E; Ganyu: 34°54′10″N, 118°59′32″E).

### Inoculation Assays

Conidia of *Magnaporthe oryzae* strains 248, 235, 162, and Guy11 were used for inoculation assays. Two-week-old Nip rice plants were used for inoculation with *M. oryzae* strains 248, 235, and 162 (all three strains are compatible). Concentration of conidia suspensions were adjusted to 5 × 10^5^ spores/mL with.2% (w/v) gelatin solution. Then, 5 ml conidia suspensions were sprayed onto leaves of inoculated plants, which were kept in a dark chamber at 85% humidity and 28°C for the first 24 h. After 24 hpi, fungal-inoculated rice seedlings were moved to a growth chamber with the same conditions as that of the greenhouse. Leaves at 8, 24, 48, 72, and 96 hpi were harvested for transcriptome sequencing and qRT-PCR assays. Two-week-old rice plants of ZH11, *OsUSP3*^OX^, *OsUSP4*^OX^, and *OsUSP5*^OX^ were used for inoculation with *M. oryzae* Guy11 according to the method above. Leaves were harvested for disease severity assessment at 6 days after inoculation.

### RNA Isolation and Illumina Sequencing

Leaves of un-incubated Nip plants were used as control samples and leaves of incubated Nip plants at 8, 24, 48, 72, and 96 hpi were used as treatment samples. Total RNA was isolated from control and treatment samples using Qiagen RNAeasy Mini kit (Qiagen Inc., Valencia, CA, United States) according to the manufacturer’s protocol. Isolated RNA was analyzed for its quality by gel electrophoresis and quantified by spectrophotometer (Nano-Drop 2000, Thermo Fisher Scientific, Wilmington, DE, United States). RNA integrity number (RIN) was calculated by using Agilent 2100 Bioanalyzer (Agilent Technologies, Thermo Fisher Scientific Inc., Waltham, MA, United States) RNA samples with RIN greater than or equal to 7 were used for library and cDNA preparation. The fragment library for RNA sequencing was prepared using Illumina True-Seq RNA Library Prep Kit (San Diego, CA, United States) according to the manufacturer’s protocol. Illumina HiSeq 2000 platform was used to generate large amounts of sequencing data performing paired-end sequencing runs using 1 g of high-quality total RNA (RIN > 7) to obtain 150 bp sequence length reads. The RNA sequencing data are deposited at the SRA website, accession numbers SRP324816 and SRP324897.

### Generation of *OsUSP3*^OX^, *OsUSP4*^OX,^ and *OsUSP5*^OX^ Transgenic Lines

The coding sequences of *OsUSP3* (LOC_Os03g19270), *OsUSP4* (LOC_Os07g36600), and *OsUSP5* (LOC_Os01g32780) were amplified using cDNA isolated from 2-week-old ZH11 as PCR templates. The amplified coding sequences were cloned into the rice transformation PXQ vector. The final construct *PXQ::OsUSP3*, *PXQ::OsUSP4*, and *PXQ::OsUSP5* were transformed into ZH11 by *Agrobacterium* (strain EHA105)-mediated co-cultivation. Transgenic plants were selected on growth media containing hygromycin (40 mg/L).

### Normalization of Expression Levels and Detection of Differentially Expressed Genes

FastQC^3^ was used to assess read quality. Reads with contaminant primer/adapters and long stretches of poor-quality bases were removed. Clean reads were mapped to the reference genomes of rice and *M. oryzae*, respectively (rice: MSU Rice Genome Annotation Project Release 7^4^; *M. oryzae*: *M. oryzae* 70-15 v3.0^5^) using TopHat version 2.1.1^6^ with default parameters (Trapnell et al., 2009). Calculation of raw read count and normalization to fragments per kilobase per million (FPKM) were performed using Cufflinks version 2.2.1 (Trapnell et al., 2012). Due to no replicate of each RNA-Seq sample, we applied the R-package DEseq^7^ to detect DEGs, following the parameters of Anders and Huber (2010). Genes with a combination of FDR≦0.01 and the absolute value of log2 (fold change) ≧1 were regarded as DEGs.

### Additional Bioinformatic Methods

The multiple alignment analysis was performed with MUSCLE version 3.8.31^8^ (Edgar, 2004). The maximum-likelihood phylogeny trees were constructed with IQ-TREE version 1.6.12^9^ (Nguyen et al., 2015) with 1,000 bootstrap values. OmicShare tools^10^ was used for enrichment analysis of GO and KEGG pathways. Prediction of secondary metabolites in rice and *M. oryzae* were performed with antismash version 5^11^ with default settings. The TBtools kit^12^ (Chen et al., 2018) was used to visualize the genome location of rice CDEGs associated with terpene biosynthesis.

### Validation of Gene Expression Using Quantitative qRT-PCR

Using Qiagen RNAeasy Mini kit (Qiagen Inc., Valencia, CA, United States), RNA isolation was performed, and cDNA synthesis was carried out using the Superscript IV Reverse transcriptase cDNA synthesis kit (TB Green ^®^ Premix Ex Taq™ II)^13^ using 2 ug template RNA. All cDNA samples were diluted to 20 ng^––1^ prior to qRT-PCR. The gene expression levels were evaluated using qRT-PCR (Bio-Rad Real-Time PCR cycler using SYBG as the fluorescent dye)^14^. The actin gene of rice (*LOC_Os03g50885*) was used as an internal reference gene. Primers were designed in Primer3^15^ and the NCBI BLASTN web platform^16^ was used to check the specificity of the sequences for the genes in question, with the low complexity filter turned off. The internal reference genes list above were used to normalize the expression levels of selected candidates.

## Data Availability Statement

The original contributions presented in the study are publicly available. This data can be found here: National Center for Biotechnology Information (NCBI) BioProject database under accession number PRJNA739552 (rice-rice blast interaction data) and PRJNA739674 (conidia and mycelium of *M. oryzae* which were used as control case for *M. oryzae*).

## Author Contributions

YnL, DL, and ZQ planned and designed the research. DL and ZQ performed the experiments. DL drafted this manuscript. YD, JY, MY, RZ, HC, XP, TS, and JQ participate in isolation of *Magnaporthe oryzae* 248, 235, and 162. YnL, YuL, and ZC supervised the manuscript, whole research and provided guidance. All authors had access to the final manuscript and approved the submission of the article.

## Conflict of Interest

The authors declare that the research was conducted in the absence of any commercial or financial relationships that could be construed as a potential conflict of interest.

## Publisher’s Note

All claims expressed in this article are solely those of the authors and do not necessarily represent those of their affiliated organizations, or those of the publisher, the editors and the reviewers. Any product that may be evaluated in this article, or claim that may be made by its manufacturer, is not guaranteed or endorsed by the publisher.

## Footnotes

1
https://antismash.secondarymetabolites.org

2
http://bcb.unl.edu/dbCAN2/

3
https://anaconda.org/bioconda/fastqc

4
http://rice.uga.edu/

5
https://www.ncbi.nlm.nih.gov/genome/?term=magnaporthe.+oryzae

6
https://ccb.jhu.edu/software/tophat/downloads/

7
http://bioinfo.au.tsinghua.edu.cn/software/degseq

8
https://anaconda.org/etetoolkit/muscle

9
http://www.iqtree.org/

10
http://www.omicshare.com/tools

11
http://plantismash.secondarymetabolites.org/

12
https://github.com/CJ-Chen/TBtools/releases

13
https://www.takarabio.com/products/real-time-pcr/real-time-pcr-kits/qpcr-with-tb-green-detection/tb-green-premix-ex-taq-ii-(tli-rnase-h-plus)

14
https://www.bio-rad.com/en-us/category/real-time-pcr-systems?ID=059db09c-88a4-44ad-99f8-78635d8d54db&WT_mc_id=200911029047&WT_srch=1&WT_knsh_id=d7b619e0-b912-446a-9ab2-ff44bb3e61ac&gclid=CjwKCAiAg6yRBhBNEiwAeVyL0Lok9TxVYu6kejVBO7Bt6JWY0uu9FLcZGpT5aVHFGM3t9PcovawBChoCrUsQAvD_BwE

15
https://bioinfo.ut.ee/primer3-0.4.0/

16
https://blast.ncbi.nlm.nih.gov/Blast.cgi

## Supplementary Material

Supplementary Figure 1 MapMan overviews of metabolism display transcriptional change at 8, 24, 48, 72, and 96 hpi. CDEGs significantly upregulated (red) and downregulated (blue) inoculated leaf samples relative to the control sample are illustrated. Individual genes are represented by small squares. The scale bar displays log2-transformed fold changes.

Supplementary Figure 2 Analysis of *O. sativa* PR genes relates to CDEGs. **(A)** Sankey plot depicting association of PR subfamilies and STEM Profiles. **(B)** RNAseq Expression profiles of *O. sativa* PR genes assigned into STEM Profile 25, 26, 0, 2, and 6 of co-expression.

Supplementary Figure 3 Diseased reactions of ZH11, *OsUSP3*^OX^, *OsUSP4*^OX^, *OsUSP5*^OX^ leaves incubated by *M. oryzae* 248, 235, and 162. **(A)** Photographs showing disease reaction of indicated rice lines and races: wild-type (ZH11); overexpression transgenic line of *OsUSP4*^OX^ (PXQ4-1, PXQ4-6, PXQ4-14). **(B)** Disease lesion area was assessed by Image J. Lesions were photographed and measured or scored at 6 days post-inoculation by isolation of *M. oryzae* 248, 235, and 162. A, B, and C represent the significant differences (one-way ANOVA test, *P* < 0.01).

Supplementary Figure 4 Analysis of *O. sativa* CDEGs associated with secondary metabolites biosynthesis. **(A)** Distribution of CDEGs in different secondary metabolites biosynthesis clusters predicted by antiSMASH. **(B)** Two terpene biosynthesis clusters include CDEGs involved in terpene biosynthesis. **(C)** Visualization based on KEGG pathway annotation of terpene synthase relates to CDEGs.

Supplementary Figure 5 Alternative splicing analysis of one RCD1-SRO-TAF4 (RST) protein (LOC_Os10g42710). **(A)** The expression value of seven transcripts of LOC_Os10g42710 at control sample and 8 hpi. **(B)** Structure and Sashimi plot of LOC_Os10g42710.1 and LOC_Os10g42710.2 transcripts. The plot of domain coordinate was displayed above the transcript structure plot.

Supplementary Figure 6 Analysis of *M. oryzae* Carbohydrate-Active enzyme (CAZyme) relates to pathogenicity. **(A)** The proportion of each CAZyme subfamilies. **(B)** The count of secreted proteins is assigned to the CAZyme subfamily of AA9, GH10, GH3, CE5, and GH7. **(C)** The expression level of *MoCDIP4* and its seven homologous genes coding proteins with a signal peptide and AA9 CAZy domain.

## Abbreviations

STEM: Short Time-series Expression Miner.
