# Supplementary figures and images for: Identification of Differentially Expressed Genes Reveal Conserved Mechanisms in the Rice-Magnaporthe oryzae Interaction
Source: Front Plant Sci. 2022 Apr 5;13:723356. doi: 10.3389/fpls.2022.723356 (PMC9016282; doi:10.3389/fpls.2022.723356)

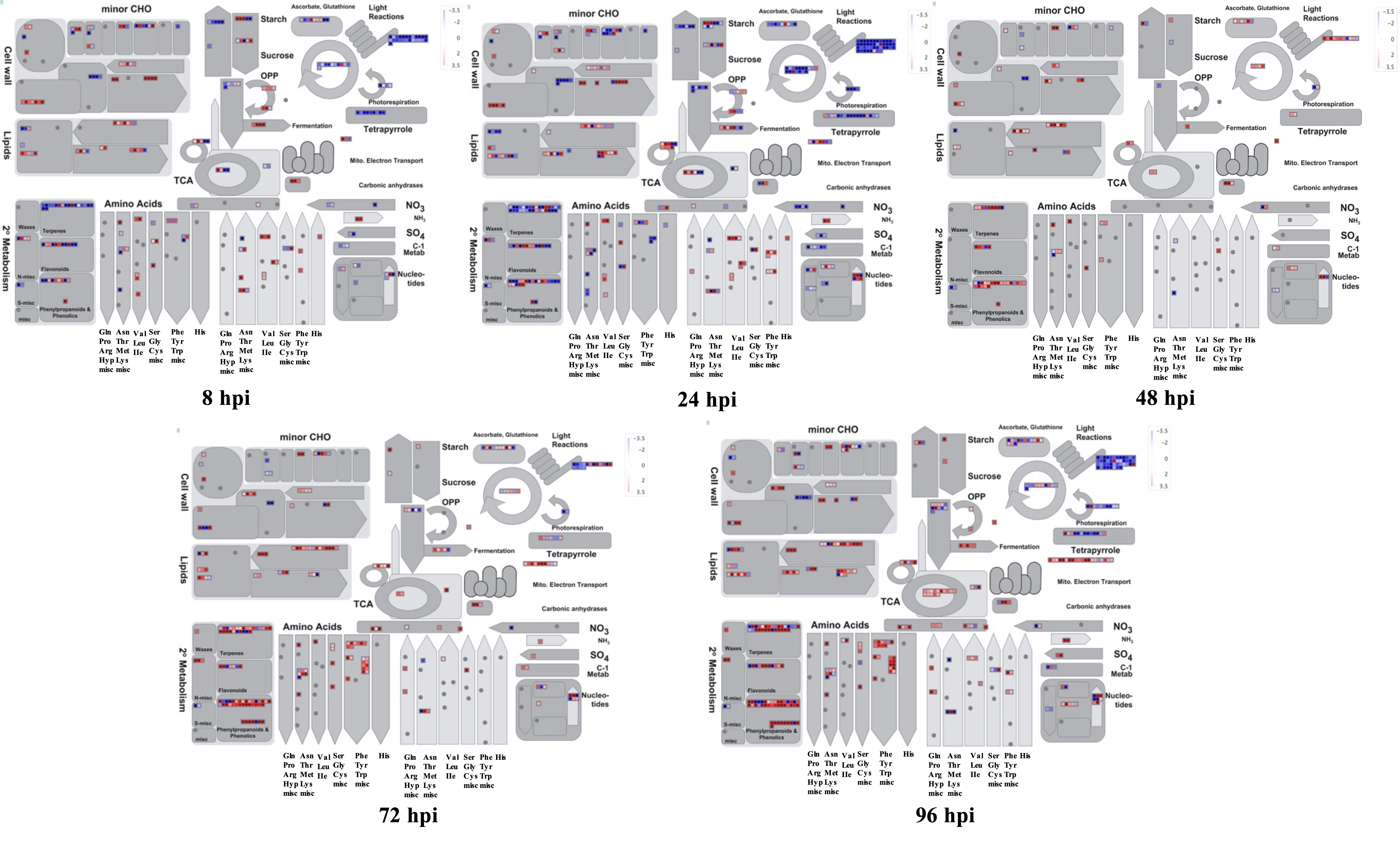

Supplement: Supplementary Figure 1 — MapMan overviews of metabolism display transcriptional change at 8, 24, 48, 72, and 96 hpi. CDEGs significantly upregulated (red) and downregulated (blue) inoculated leaf samples relative to the control sample are illustrated. Individual genes are represented by small squares. The scale bar displays log2-transformed fold changes. [file Image_1.JPEG]

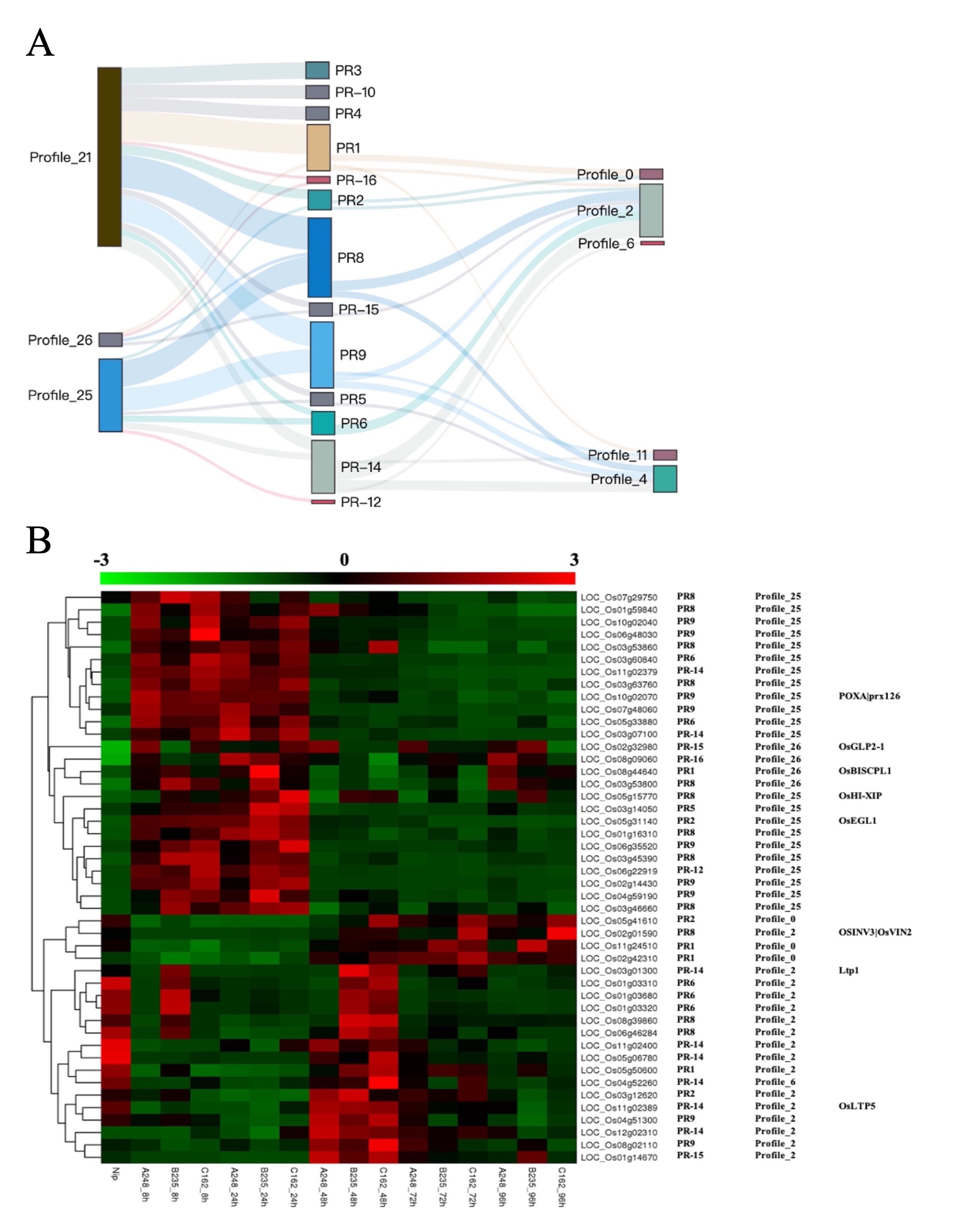

Supplement: Supplementary Figure 2 — Analysis of O. sativa PR genes relates to CDEGs. (A) Sankey plot depicting association of PR subfamilies and STEM Profiles. (B) RNAseq Expression profiles of O. sativa PR genes assigned into STEM Profile 25, 26, 0, 2, and 6 of co-expression. [file Image_2.JPEG]

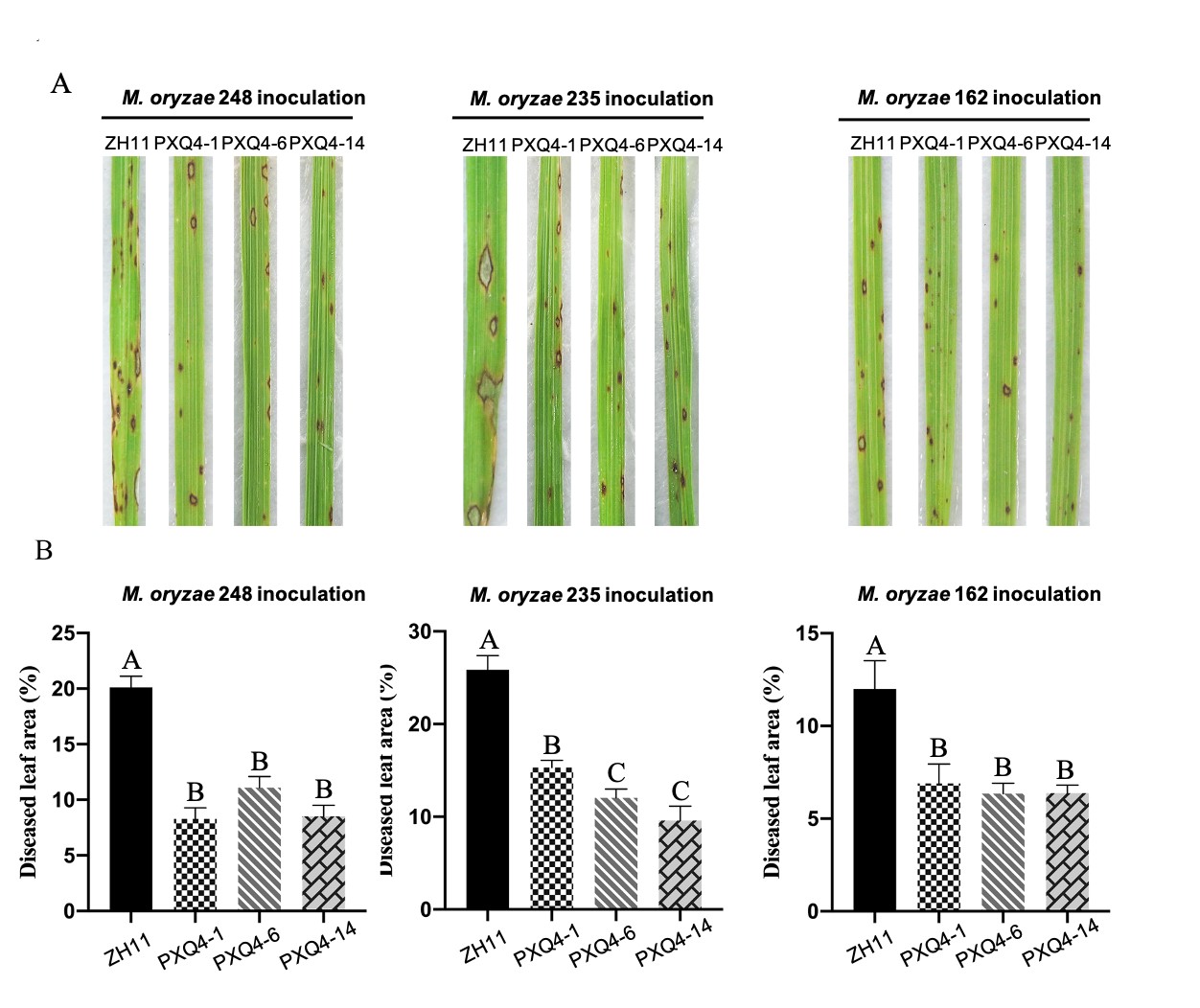

Supplement: Supplementary Figure 3 — Diseased reactions of ZH11, OsUSP3OX, OsUSP4OX, OsUSP5OX leaves incubated by M. oryzae 248, 235, and 162. (A) Photographs showing disease reaction of indicated rice lines and races: wild-type (ZH11); overexpression transgenic line of OsUSP4OX (PXQ4-1, PXQ4-6, PXQ4-14). (B) Disease lesion area was assessed by Image J. Lesions were photographed and measured or scored at 6 days post-inoculation by isolation of M. oryzae 248, 235, and 162. A, B, and C represent the significant differences (one-way ANOVA test, P < 0.01). [file Image_3.JPEG]

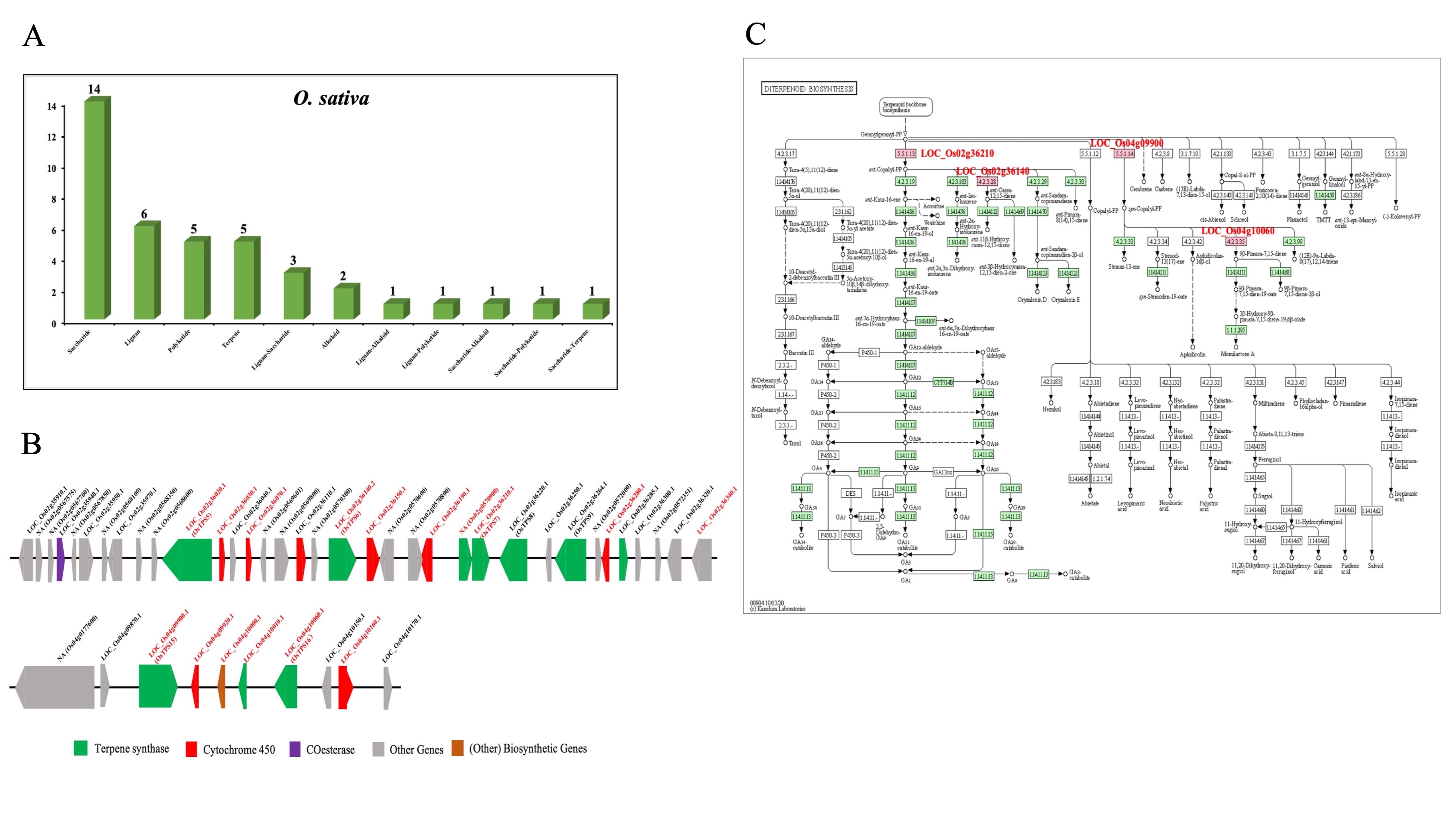

Supplement: Supplementary Figure 4 — Analysis of O. sativa CDEGs associated with secondary metabolites biosynthesis. (A) Distribution of CDEGs in different secondary metabolites biosynthesis clusters predicted by antiSMASH. (B) Two terpene biosynthesis clusters include CDEGs involved in terpene biosynthesis. (C) Visualization based on KEGG pathway annotation of terpene synthase relates to CDEGs. [file Image_4.JPEG]

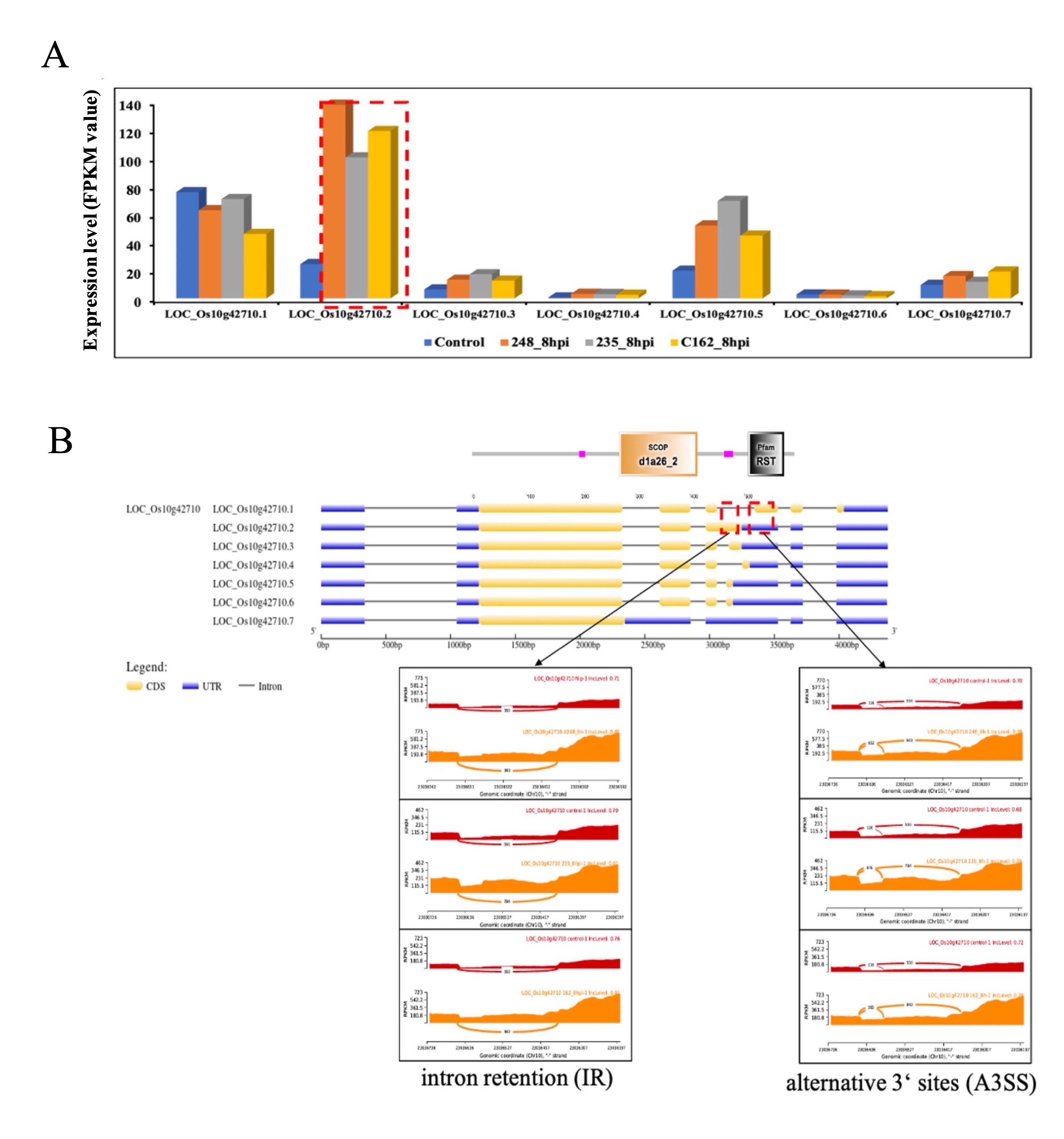

Supplement: Supplementary Figure 5 — Alternative splicing analysis of one RCD1-SRO-TAF4 (RST) protein (LOC_Os10g42710). (A) The expression value of seven transcripts of LOC_Os10g42710 at control sample and 8 hpi. (B) Structure and Sashimi plot of LOC_Os10g42710.1 and LOC_Os10g42710.2 transcripts. The plot of domain coordinate was displayed above the transcript structure plot. [file Image_5.JPEG]

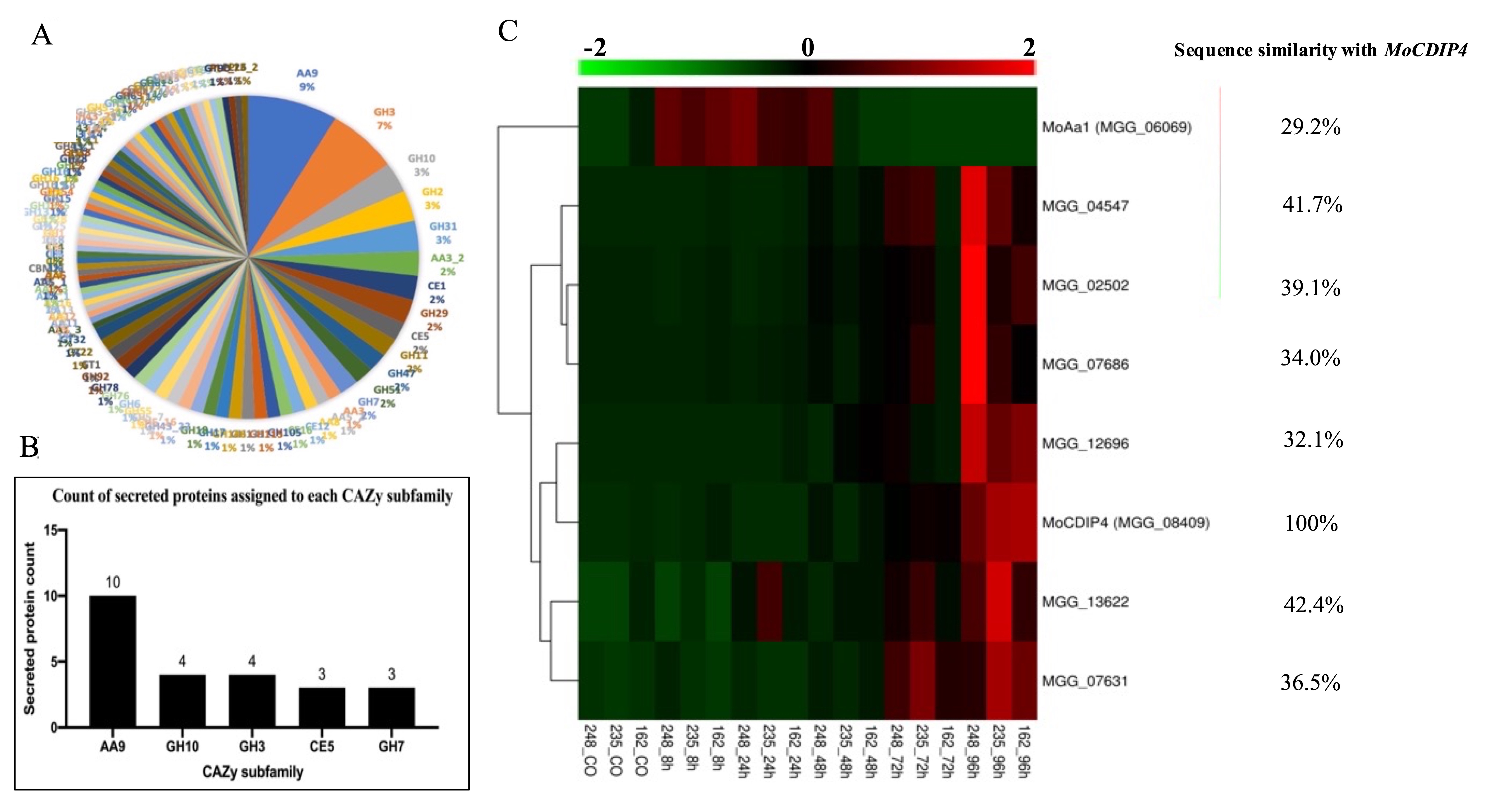

Supplement: Supplementary Figure 6 — Analysis of M. oryzae Carbohydrate-Active enzyme (CAZyme) relates to pathogenicity. (A) The proportion of each CAZyme subfamilies. (B) The count of secreted proteins is assigned to the CAZyme subfamily of AA9, GH10, GH3, CE5, and GH7. (C) The expression level of MoCDIP4 and its seven homologous genes coding proteins with a signal peptide and AA9 CAZy domain. [file Image_6.JPEG]
